# Structure‐Based Virtual Screening and *in vitro* and *in vivo* Analyses Revealed Potent Methyltransferase G9a Inhibitors as Prospective Anti‐Alzheimer's Agents

**DOI:** 10.1002/cmdc.202200002

**Published:** 2022-05-19

**Authors:** Aina Bellver‐Sanchis, Bhanwar Singh Choudhary, Júlia Companys‐Alemany, Sukanya, Pedro A. Ávila‐López, Antón Leandro Martínez Rodríguez, Jose Manuel Brea Floriani, Ruchi Malik, Mercè Pallàs, Belén Pérez, Christian Griñán‐Ferré

**Affiliations:** ^1^ Pharmacology Section Department of Pharmacology Toxicology and Therapeutic Chemistry Faculty of Pharmacy and Food Sciences Institute of Neuroscience, Universitat de Barcelona (NeuroUB) Av. Joan XXIII 27–31 08028 Barcelona Spain; ^2^ Department of Pharmacy Central University of Rajasthan Bandarsindari Ajmer 305817 India; ^3^ Department of Pharmaceutical Chemistry and Quality Assurance Shree S. K. Patel College of Pharmaceutical Education and Research Ganpat University Mehsana Gujarat 384012 India; ^4^ Department of Biochemistry and Molecular Genetics Feinberg School of Medicine Northwestern University Chicago Illinois USA; ^5^ Centro de Investigación en Medicina Molecular y Enfermedades Crónicas (CIMUS) Departamento de Farmacología Farmacia y Tecnología Farmacéutica Universidad de Santiago de Compostela 15782 Santiago de Compostela Spain; ^6^ Department of Pharmacology, Therapeutic and Toxicology Universitat Autònoma de Barcelona 08193 Barcelona Spain

**Keywords:** Alzheimer's disease, epigenetics, G9a methyltransferase, amyloid-β, structure based virtual screening

## Abstract

G9a is a lysine methyltransferase able to di‐methylate lysine 9 of histone H3, promoting the repression of genes involved in learning and memory. Novel strategies based on synthesizing epigenetic drugs could regulate gene expression through histone post‐translational modifications and effectively treat neurodegenerative diseases, like Alzheimer's disease (AD). Here, potential G9a inhibitors were identified using a structure‐based virtual screening against G9a, followed by *in vitro* and *in vivo* screenings. First, screening methods with the AD transgenic *Caenorhabditis elegans* strain CL2006, showed that the toxicity/function range was safe and recovered age‐dependent paralysis. Likewise, we demonstrated that the best candidates direct target G9a by reducing H3 K9me2 in the CL2006 strain. Further characterization of these compounds involved the assessment of the blood‐brain barrier‐permeability and impact on amyloid‐β aggregation, showing promising results. Thus, we present a G9a inhibitor candidate, **F**, with a novel and potent structure, providing both leads in G9a inhibitor design and demonstrating their participation in reducing AD pathology.

## Graphical abstract

**Epigenetic modifications**: A structure‐based virtual screen led to the identification of new scaffolds as potent G9a inhibitors. These promising hits exhibited better *in silico* profiles, IC_50_ values, and BBB permeability than currently published inhibitors for G9a. The best G9a inhibitor candidates directly target G9a, decreasing the levels of the repressive mark H3 K9me2 (demethylation of H3 K9), and beneficial effects on amyloid aggregation were observed in *C. elegans*.

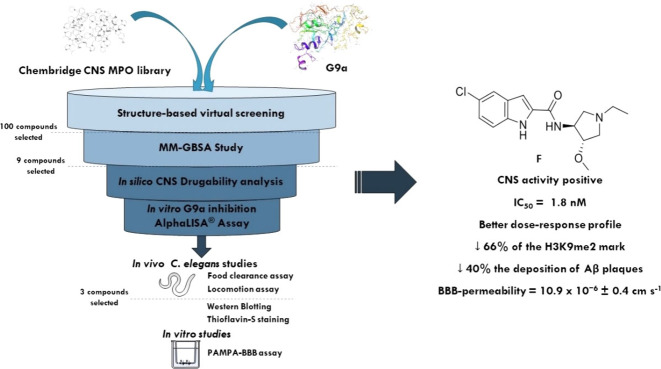

## Introduction

Epigenetic modifications have an important impact on regulating the expression and transcription of genes involved in many human diseases, such as cancer,^[1]^ addiction,^[2]^ psychiatric^[3]^ and neurodegenerative disorders.^[4]^ One of the most relevant epigenetic mechanisms to control gene expression is chromatin remodeling.^[5]^ This is catalyzed by different enzymes and is responsible for the dynamic modification of chromatin structure, enabling or restricting the access of the transcription machinery to condensed genomic DNA. An essential process responsible for the alteration of chromatin architecture is the post‐translational modification (PTM) of histones. PTMs are chemical modifications of the amino acid residues of proteins, leading to changes in gene expression. This process is mainly mediated by epigenetic “writer” enzymes, such as histone methyltransferases (HMTs), which are responsible for adding chemical methyl groups in specific residues (lysine (K) or arginine (R)), and as a consequence, leading to active or repressive marks. In general, methylation of H3 K9 and H3 K27 are typical hallmarks of transcription repression, whereas methylation of H3 K4, H3 K36, and H3 K7 are considered active marks. Hence, dysregulation of methylation at specific histone sites has been implicated in many human diseases, highlighting the potential therapeutic value of HMTs as targets.^[6]^

In particular, G9a methyltransferase, also known as euchromatic histone methyltransferase 2 (EHMT2), is an HMT responsible for mono‐ and di‐methylate lysine 9 on histone H3 (H3 K9me1 and H3 K9me2).^[7]^ Consequently, these repressive marks are capable of regulating gene expression.^[8]^ Until now, G9a has been widely explored as an anti‐cancer and an anti‐malarial target.[^9^, ^10^, ^11^] However, interestingly, recent evidence potentially suggests that G9a is a target in neurodegenerative disorders, such as Alzheimer's disease (AD), since its epigenetic marks are related to the repression of transcription in genes involved in learning and memory formation, contributing to cognitive impairment.[^12^, ^13^, ^14^]

**Figure 1:**
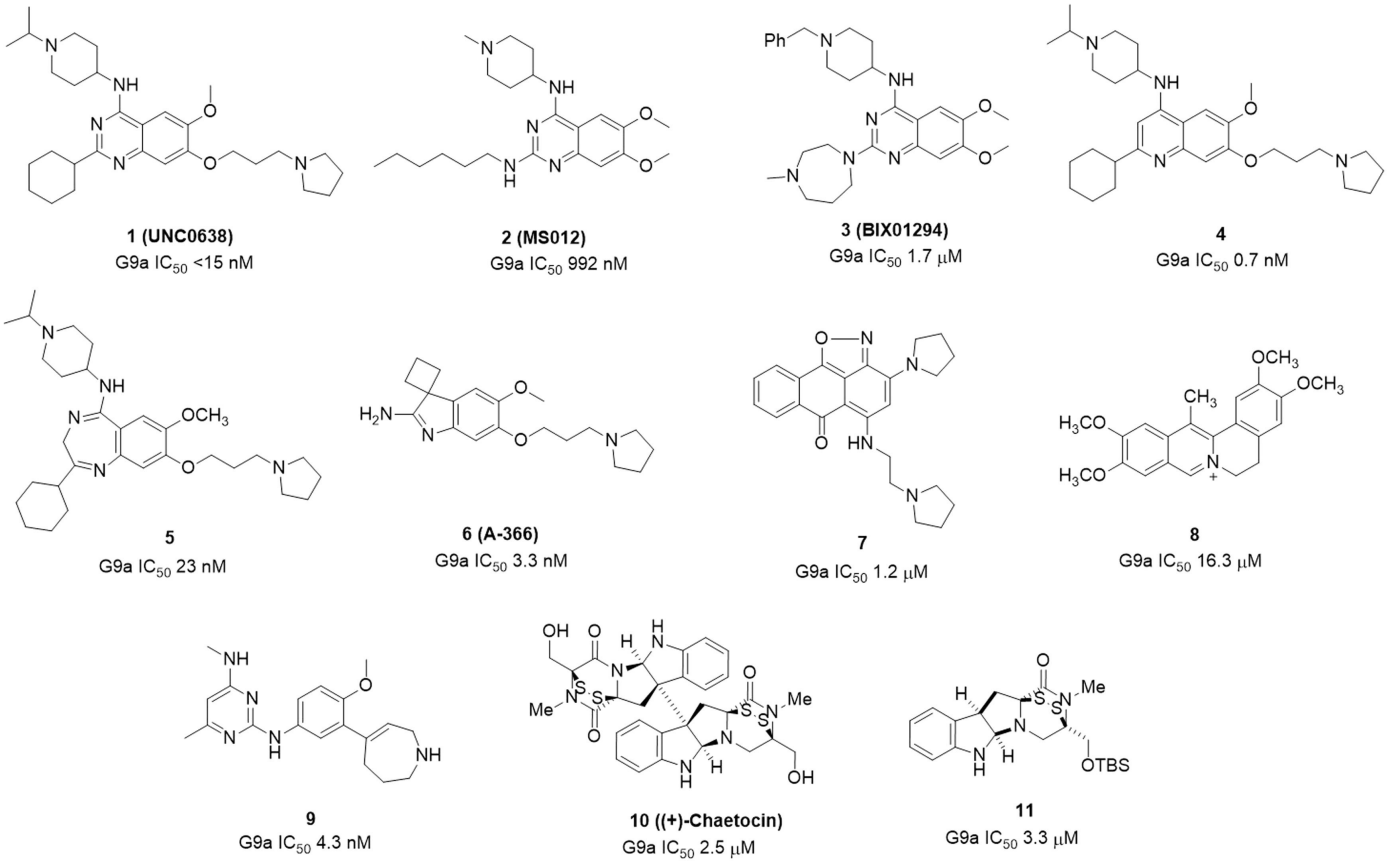
Structures of previously reported G9a inhibitors (IC_50_ values are dependent on the assay conditions used).

Small molecule inhibitors of G9a are valuable tools to study this protein's biological functions and further explore the therapeutic hypotheses associated with them, for instance, in the context of neurodegeneration. Several G9a inhibitors have been synthesized and published in the last decade (Figure 1). Among the previously reported G9a inhibitors, quinazolines (1,2,3)[^9^, ^10^] and quinolines (4)[^15^, ^16^] are widely explored; others are benzodiazepines (5),^[17]^ amino‐indoles (6),^[18]^ 6H‐anthra[1,9‐cd]isoxazol‐6‐one (7),^[19]^ pseudodehydrocorydaline (8),^[20]^ 2,4‐diamino‐6‐methylpyrimidines (9),^[21]^ Chaetocin (10)[^22^, ^23^, ^24^] and Epidithiodiketopiperazine (11).^[25]^ The first G9a inhibitor identified by high‐throughput screening (HTS) was BIX01294 (Figure 1), which resulted in active cell‐based assay and reduced H3 K9methylation levels in several G9a target genes. However, this compound had a toxicity/function ratio lower than 6 (determined by dividing the half‐maximal effective concentration (EC50) value of observed toxicity by the half‐maximal inhibitory concentration (IC_50_) value of the functional potency), so to overcome its cytotoxicity, more specific and potent molecules need to be discovered.[^26^, ^27^] Crystallographic studies based on BIX01294 identified other inhibitory compounds to improve the therapeutic range. Further optimization of UNC0638 led to potent and selective G9a inhibitors with a better toxicity/function ratio than BIX01294 (>100). However, these two and the other published compounds were less effective in *in vitro* and *in vivo* studies and had poor blood‐brain barrier (BBB) permeability and very high melting points. Thus, there is a need to explore new chemical scaffolds other than quinazolines and quinolines, which exhibit improved pharmacokinetic properties.

In brief, this work is based on the hypothesis that overexpression of G9a and its repressive mark serve as a driver of repression of genes involved in learning and memory. Therefore, we explored an extensive chemical database (Chembridge CNS MPO database) using structure‐based virtual screening against G9a, to provide potent inhibitors with novel chemical scaffolds as a research tool to study the function of this protein in biological processes, mainly focused on AD.

## Results and Discussion

### Identification of novel G9a inhibitors through structure‐based virtual screening

**Figure 2:**
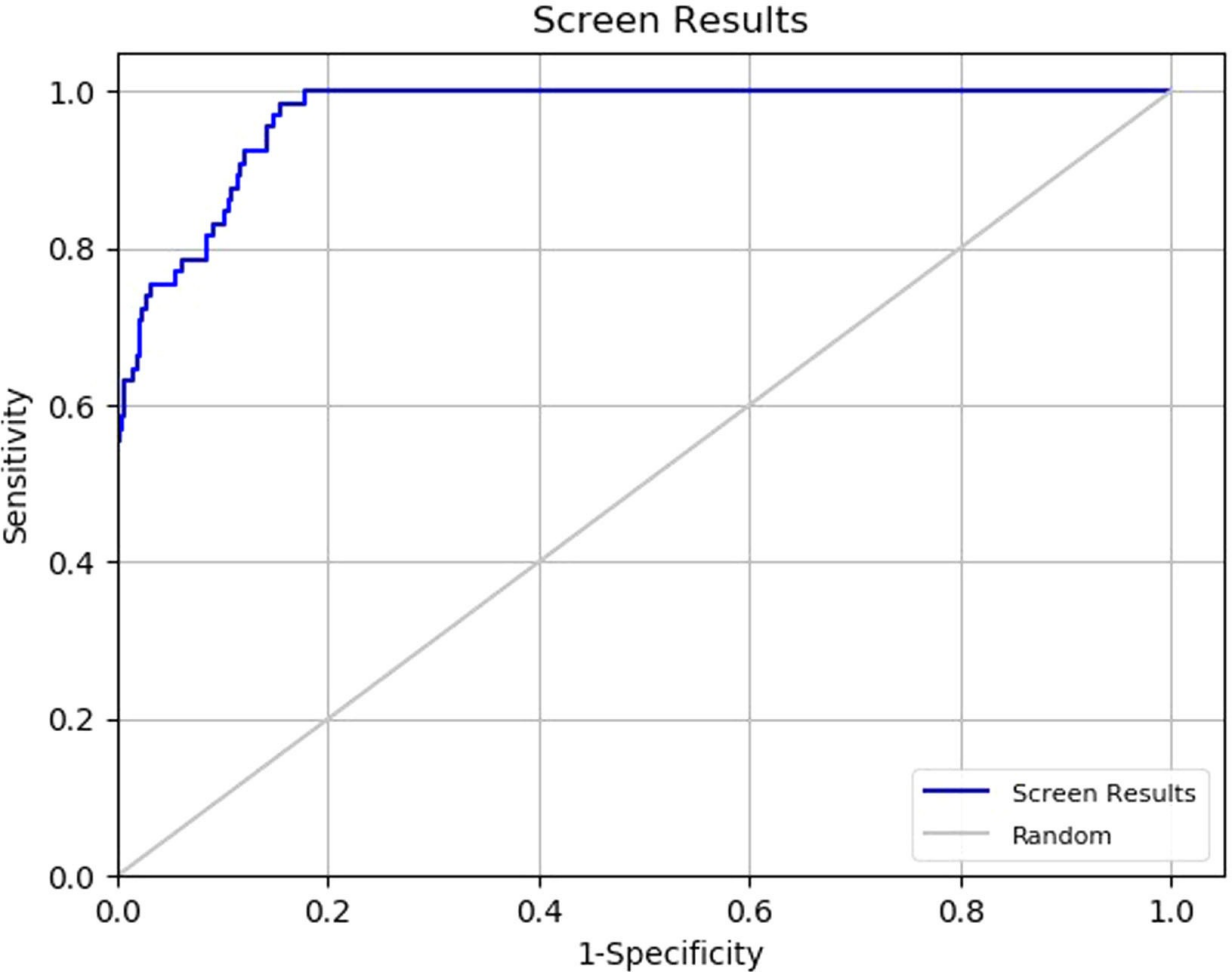
ROC graphs of the docking results for assessing the reliability of the docking protocol.

Protein Data Bank (PDB) id 5TTF is selected for creating a docking model based on its 1.72 Å resolution, the sequence length of 283 amino acids, no Ramachandran outliers, and quinazoline derivative MS012 (Figure 1) as a co‐crystallized compound. The crystal structure of G9a bound to MS012 (PDB ID: 5TTF) was retrieved from the RCSB portal and prepared using the protein preparation wizard of Maestro. The enrichment‐based validation of the docking protocol was performed, showing the receiver operating characteristic (ROC) graph where docking results were plotted between valid positive rate (sensitivity) and false‐positive rate (1‐specificity). The ROC graph reflects the probability of a higher ranking of randomly selected active compounds over randomly selected decoy molecules. The results of Glide SP docking showed ROC values of 0.97 for 5TTF (Figure 2). The RMSD of co‐crystallized and docked MS012 was found 1.165 Å (Figure S1a).

**Figure 3:**
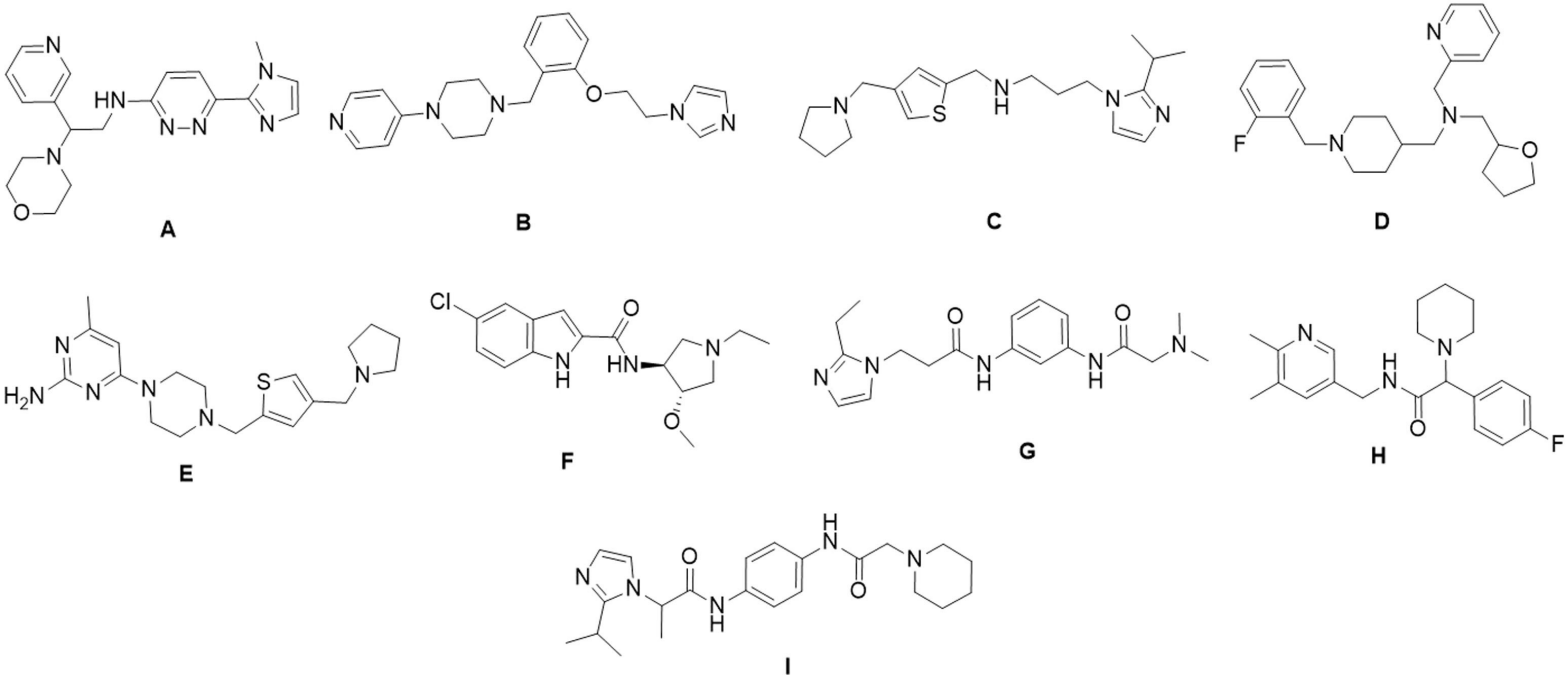
Structure of virtually screened and selected molecules.

A hierarchical stepwise virtual screening was performed using Glide HTVS, SP, and XP docking for the Chembridge database of 496644 compounds and XP docking of UNC0638 as a reference compound. The top 100 compounds were selected, based on docking scores from the central nervous system multiparameter optimization (CNS MPO) database of Chembridge Corporation, for further analysis. The docking scores of the top 100 compounds ranged from −13.592 kcal/mol to −10.105 kcal/mol. The Molecular Mechanics‐Generalized Born Surface Area (MM‐GBSA) dG binding affinity of these 100 compounds ranged from −116.98 kcal/mol to −51.09 kcal/mol. 9 molecules were selected based on the Glide XP dock score, MM‐GBSA dG binding affinity, hydrogen bond mediated interaction with G9a active residues, i. e. Aspartate (Asp)1074, Asp1083, Leucine (Leu)1086, Asp1088, and hydrophobic interaction with Tyrosine (Tyr)1154, and Phenylalanine (Phe)1158[^9^, ^28^, ^29^, ^30^] and scaffold novelty (Figure 3a, 5, S5, Table 1 and Table S1). These selected molecules were further subjected to Pan‐assay interference compounds (PAINS) filter and all were found to be non‐PAINS.^[31]^

**Table 1:** Dock score, MM‐GBSA dG score, interacting residues, and G9a IC_50_ (nM) of selected screened molecules.

Compound	Dock Score	MM‐GBSA dG	G9a IC_50_ [nM]
**UNC0638**	−5.049	−70.31	9.4
**A**	−10.346	−106.68	>100
**B**	−12.553	−116.98	9.4
**C**	−10.809	−90.21	1.7
**D**	−10.552	−101.76	10.5
**E**	−11.548	−106.01	2.1
**F**	−10.202	−95.02	1.8
**G**	−10.487	−93.42	5.8
**H**	−10.331	−96.43	2.5
**I**	−11.253	−91.44	3.9

### In vitro G9a inhibition assay

The percentages of G9a activity for the 10 selected molecules were determined at 100 μM S‐adenosylmethionine (SAM) and peptide substrate using AlphaLISA® technology. These percentages were used to calculate the IC_50_. As a control, we used the well‐established G9a inhibitor, **UNC0638**. The IC_50_ against G9a of the UNC0638 was 9.4 nM, in accordance with reported studies that have described an IC_50_<15 nM. No significant inhibition was observed by A, and it was therefore considered as not active (IC_50_>100 nM). Meanwhile, **D** presented higher IC_50_ in comparison with either **UNC0638** or the other compounds (IC_50_=10.5 nM), and therefore these compounds were excluded for further studies. Compound **B** reached an IC_50_ equal to that of **UNC0638** (IC_50_=9.4 nM). The rest of compounds were at least 4‐fold more potent as G9a inhibitors than **UNC0638** (IC_50_ **C**=1.7 nM; IC_50_ **E**=2.1 nM; IC_50_ **F**=1.8 nM; IC_50_ **G**=5.8 nM; IC_50_ **H**=2.5 nM; IC_50_ **I**=3.9 nM) (Table 1, Figure S2 and Table S2).

### In silico ADMET analysis

Moreover, physicochemical descriptors and pharmaceutically relevant properties of the selected compounds were calculated with Schrödinger QikProp,^[32]^ an ADME program useful for predicting the CNS‐relevant drug properties. For instance, CNS activity was predicted using a scale, with the highest activity for those that scored +2, as was the case for **E**, **F**, **G**, and **I**; otherwise, the lowest activity was presented by **B**, **D**, with 0 as their score. Aqueous solubility (logS) could significantly affect the compounds’ absorption and distribution. All selected compounds presented a similar value, fitting the recommended range (−6.5–0.5). Other properties calculated were total solvent accessible surface area (SASA), octanol/water partition coefficient (logPo/w), apparent Caco‐2 cell permeability (PCaco), brain/blood partition coefficient, apparent MDCK cell permeability (PMDCK), skin permeability (logKp), binding to human serum albumin (logKhsa), Van der Waals surface area of polar atoms (PSA) and the number of Lipinski's rule of five violations. Moreover, human oral absorption (HOA) and the percent of HOA (%HOA) were predicted, resulting high in all of them, although the %HOA of **E** was not strictly superior to 80 %. Additionally, hydrogen bond acceptor and donor (HBA, and HBD, respectively). A total of 3 compounds **A** (HBA=9.2), **G** (HBA=8.5), and **I** (HBA=8.5) were not able to pass the qualifying limit for the number of HBA. UNC0638 is can also not pass the qualifying limit for SASA, logKhsa, and Lipinski rule of 5. To balance these properties, 6 compounds that showed CNS drug properties were considered potential hit compounds as drug candidates (Table S3).

#### In vivo efficacy studies in Caenorhabditis elegans (C. elegans)

Keeping in mind that our main goal in this work is to provide new and potent chemical scaffolds to treat AD pathology, we proposed to use the *C. elegans* model to evaluate their effect on amyloid‐β (Aβ)‐mediated neurodegeneration. First, food consumption by the *C. elegans* strain N2 (Wild‐Type) (N2 (WT)) was assessed by the variation in the optical density (OD_595_) of bacteria over time as a measure of compounds toxicity, expecting a normal development and well‐being of the animals in each dose tested. Although **UNC0638** is a published compound described as a non‐toxic compound in *in vivo* studies, we also used it as a control in the assay (Figure S3). The OD_595_ of all compounds at different concentrations was not different from the respective OD_595_ of the negative control in a statistically significant manner (Figure S3). Thus, all of them were classified as safe, as the OD_595_ decrease was parallel to that of the negative control samples, and visual inspection confirmed the average growth of the animals.

#### Locomotion assay

**Figure 4:**
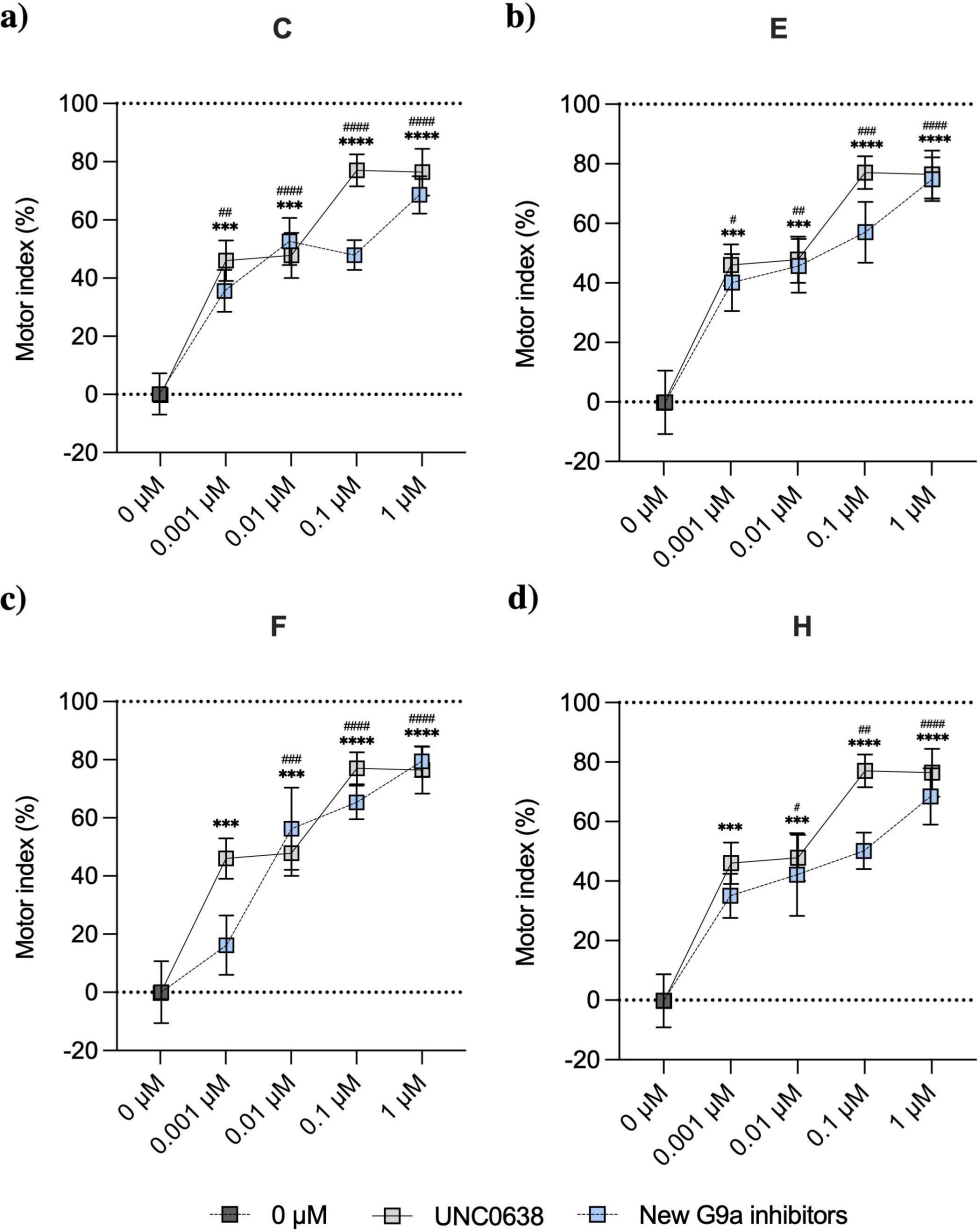
a‐d) Treatment with G9a inhibitors ameliorates motor dysfunction of CL2006 worms. For dose‐response: Values represented are mean ± Standard error of the mean (SEM); n=3 with at least 90–100 worms in each group. Statistical analysis: One‐Way ANOVA, followed by Dunnett post‐hoc analysis. 0 μM Vs. UNC0638 treatment: ***p<0.001; ****p<0.0001. 0 μM Vs. new G9a inhibitor treatment: #p<0.05; ##p<0.01; ###p<0.001; ####p<0.0001.

One of the best‐characterized transgenic AD strains in *C. elegans* is the CL2006 strain, which contains the Punc‐54::Aβ_1–42_ transgene, showing a progressive adult‐onset paralysis. Then, to establish a dose‐response profile, the pharmacological impact of G9a inhibition was evaluated in a locomotion assay with this *C. elegans* strain. We performed a motility assay where animals were placed in the center of a seeded plate and allowed to explore the environment freely. After 1 min, the animals that did not cross 1 cm circumference were scored as motor defective. N2 (WT), and CL2006 strain were treated with dimethyl sulfoxide (DMSO) 1 %, used as a vehicle, which should not cause a relevant locomotor defective phenotype. As expected, the percentage of locomotion defects in N2 (WT) animals was the lowest (∼16 %); whereas, as a result of the age‐dependent paralysis, CL2006 strain showed a significant percentage of defects in their motility (∼60 %) (Figure S4a), verifying that locomotor behavior is statistically different between the two strains of *C. elegans* allowing us to study the impact of G9a inhibitors after chronic treatment. The motor index for each condition was calculated using the motor performance of Bristol (N2) (WT, 100 %) and the transgenic AD strain (CL2006, 0 %) animals as reference. The well‐establishing inhibitor, **UNC0638**, was tested and used as a control compared to the other selected compounds (Figure 4).

**Figure 5:**
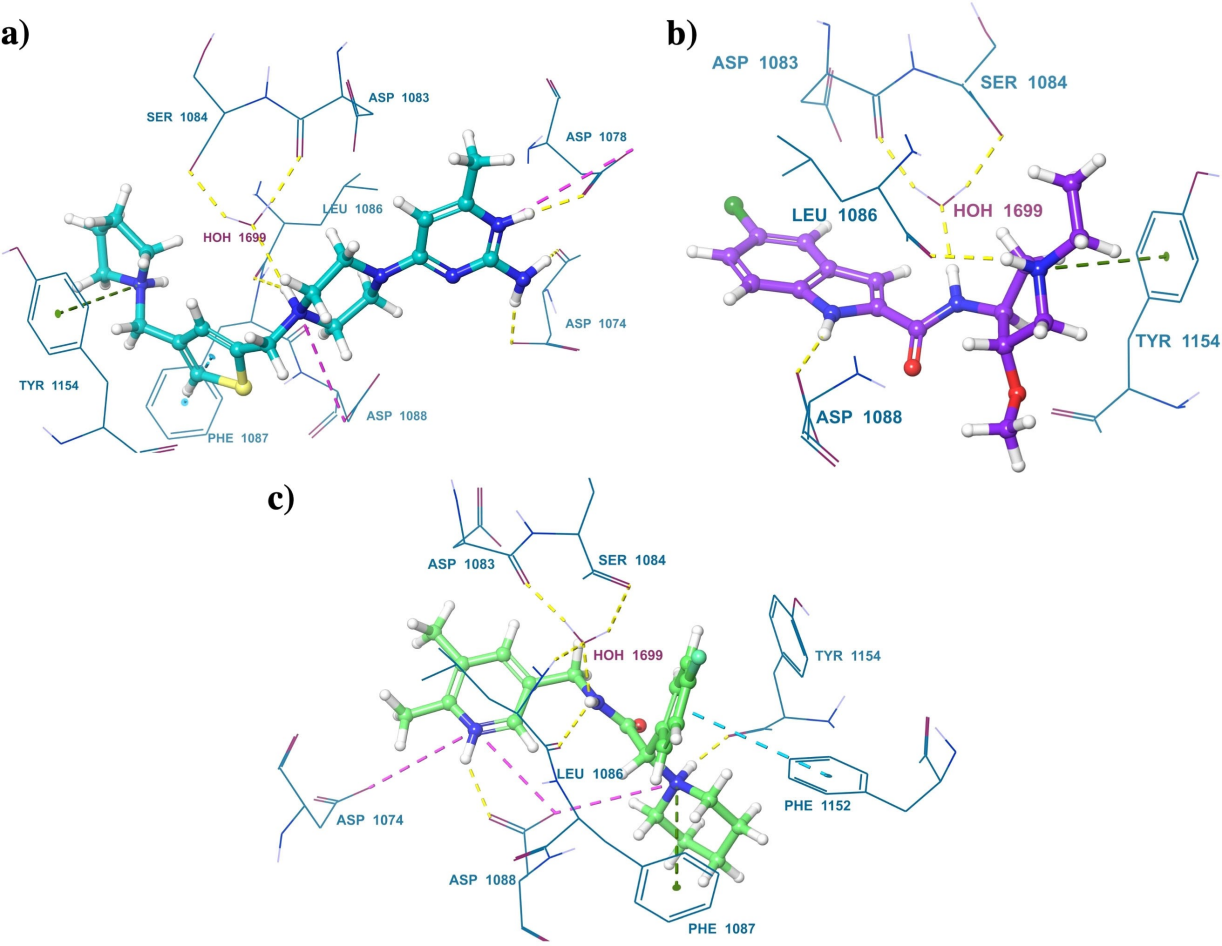
3D interactions diagram of a) E−G9a, b) F−G9a, and c) H−G9a. Protein‐ligand Interactions were denoted by a dotted line. Hydrogen bond: Yellow; Salt bridge: Pink; π‐π stacking: blue; π‐cation: Green.

By correlating *in silico* and *in vitro* G9a activity results, it is proved that interactions mediated by HBD, water bridge (WB), salt bridge (SB), and π‐cation (π‐cat) with the amino acid residues Asp1074, Asp1083, Asp 1084, Leu1086, Asp1088, and Tyr1154 are essential for the potent activity of G9a inhibitors. The pyrrolidine, morpholine, oxazepane, piperidine, piperazine, imidazole, pyridine, pyrimidine, indole, etc. moieties were present in the screened moiety. Nitrogen atoms present in these moieties interacted with G9a through a hydrogen bond, salt bridge, and π‐cation interactions. These moieties interacted with Asp1083, Asp1084, Asp1086, and Asp1088 (Figure 5, Figure S5). Although some of these interactions were present in compound **B**, the dose‐response profile did not show an improved performance against the motor indexes of the control **UNC0638** (Figure S4b). Moreover, as aforementioned, we described that both IC_50_ were very similar, and it did not present better properties among the selected compounds, such as the CNS activity, justifying its exclusion from the study. NH of amide and 2° amine group in **C**, **F**, **G**, **H**, and **I** are equally important and responsible for interaction with Asp1083, Asp1086, and Asp1088. In parallel fashion, **E** showed SB interaction with the Asp1088, WB interaction with the Asp1083, and HBD interaction with Leu1086 being essential interactions for potent inhibitory activity of G9a. The dose‐response profile of **I** showed the lowest motor index in the maximum and minimum dose tested, and in the case of **G** the motor index decreased progressively as the dose decreased (Figure S4c,d). However, due to **G** and **I** not passing the qualifying limit of all of the CNS drug properties described above, these compounds were discarded. **C**, **E**, **F**, and **H** motor index decreased progressively with the doses tested, ameliorating the paralysis presented by CL2006. Moreover, the motor indexes of each dose were not statistically different from the profile of the control UNC0638 (Figure 4a–d). The NH of amide group interacted with water bridge mediated hydrogen bond donor interaction with Asp1083 and Ser1084, NH of indole ring interacted through hydrogen bond donor interaction with Asp1088 of **F**; such interaction is not present in other compounds, and possibly because of this interaction, **F** is the most potent compound with an IC_50_ of 1.8 nM (Figure 5c, Figure S2e). In the same way, the motor index of **F** decreased progressively with the doses tested.

#### Effects of the best G9a inhibitor candidates on the repressive mark H3 K9me2

**Figure 6:**
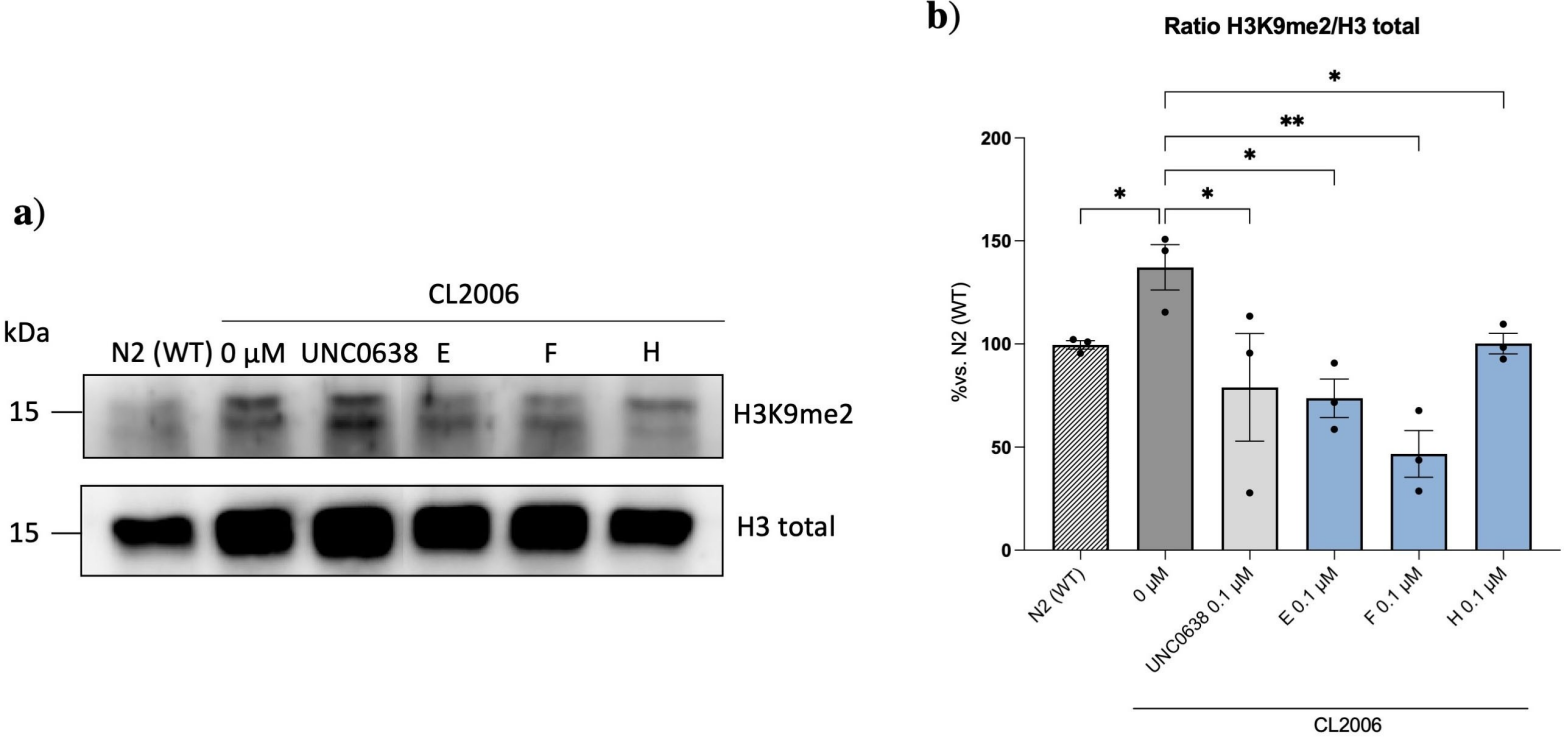
a‐b) Representation of western blotting and quantification of the total H3 K9me/H3 ratio after treatments with the best candidates to inhibit G9a. Values represented are mean ± Standard error of the mean (SEM); n=3 with at least 200 worms in each group. Statistical analysis: One‐Way ANOVA, followed by Dunnett post‐hoc analysis: 0 μM Vs. UNC0638, E, and F treatment: *p<0.05;**p<0.01. Statistical analysis: Unpaired t‐test: N2 (WT) Vs. 0 μM: *p<0.05; 0 μM Vs. H: *p<0.05.

Considering the results described heretofore, we selected four compounds to go deeper into their effects on AD pathology. Firstly, to demonstrate that these compounds directly target G9a, we quantified the levels of its repressive mark H3 K9me2 after each drug treatment (Figure 6). First, we compared the ratio H3 K9me2/H3 total levels in the N2 (WT) strain compared to the AD transgenic strain (CL2006). Interestingly, the CL2006 presented higher levels of H3 K9me2, confirming the link between this repressive mark and AD pathology. Regarding each compound, we observed that the ratio H3 K9me2/H3 total levels after each drug intervention were reduced, suggesting that our compounds target G9a directly (Figure 6b).

#### Inhibition of Aβ aggregation in C. elegans

**Figure 7:**
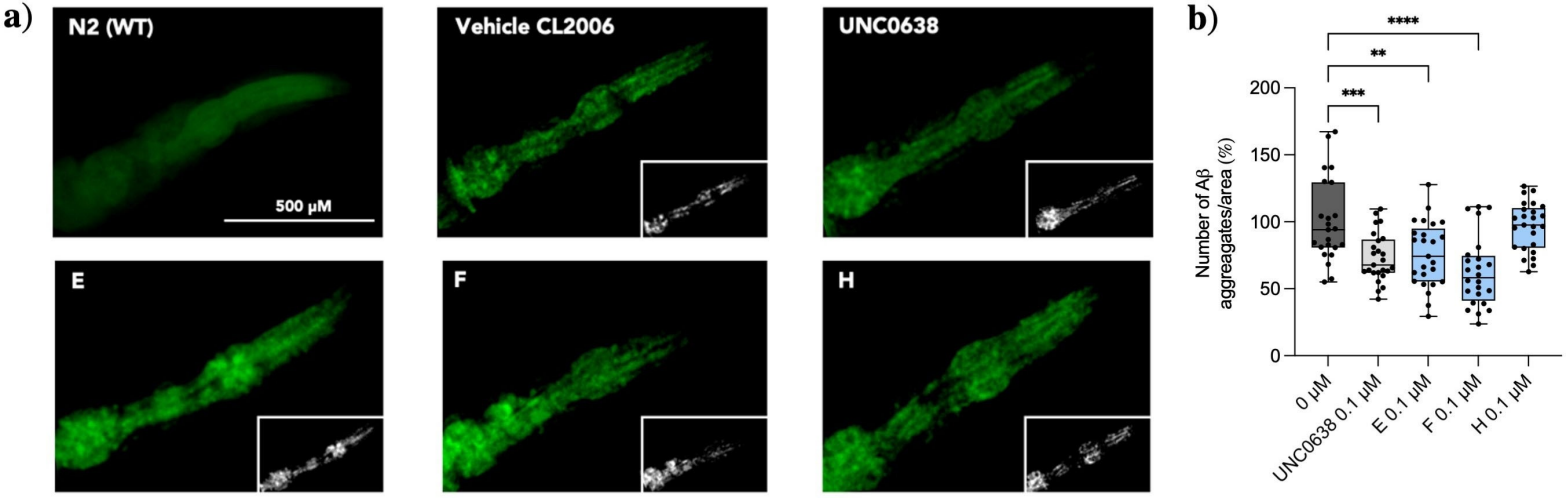
a) Representative images from each group tested. b) Quantification of Thioflavin S‐positive particles in the head region of CL2006 strain. Values represented are mean ± Standard error of the mean (SEM); n=3 with 25 worms in each group. Statistical analysis: One‐Way ANOVA, followed by Dunnett post‐hoc analysis. Vehicle Vs. new G9a inhibitor treatment: **p<0.001; ***p<0.001; ****p<0.0001.

Furthermore, we evaluated the impact after the pharmacological inhibition of G9a treatment on the deposition of Aβ aggregates, as an important hallmark of AD, using Thioflavin‐S (ThS) staining. The transgenic AD strain CL2006 is characterized by the expression of human Aβ_1‐42_ under the control of a muscle‐specific promoter, making it suitable to answer this objective in our work. As we mentioned above, **UNC0638** was used as a positive control. Thus, as expected, **UNC0638** reduced the Aβ deposition by approximately 25 %. For the four proposed candidates from our tested library, all of them showed the same tendency but in a statistically different way. Similarly, in **UNC0638**, the reduction in the number of deposits by compound **E** was approximately 25 %. **F** reduced Aβ aggregation by approximately 40 % compared to the vehicle group, being more effective in reducing Aβ aggregation than UNC0638. Unfortunately, treatment with **H** could not alleviate the aggregation of Aβ plaques (3 %), although previous *in silico* and *in vitro* results were promising (Figure 7).

### In vitro BBB penetration study

Then, we performed the parallel artificial membrane permeation assay (PAMPA) to predict BBB penetration (PAMPA‐BBB), obtaining important information for compound selection and synthesis guidance for desirable CNS properties. The selected compounds **E**, **F**, and **H** had *in vitro* permeability (P_e_) values of 2.2×10^−6^±0.1 cm ⋅ s^−1^, 10.9×10^−6^±0.4 cm ⋅ s^−1^ and 17.4×10^−6^ ±2.4 cm ⋅ s^−1^, respectively (Table 2). The compound **E** had lower P_e_ compromising its permeability, being lower than the P_e_ value of the control **UNC0638** (PAMPA−BBB P_e_=6.2×10^−6^ cm ⋅ s^−1^). Nevertheless, the compounds **F** and **H** presented positive BBB permeation, being able to cross the BBB and reach their biological target in the CNS. Therefore, the compound **F** is a promising tool for *in vivo* AD studies with mammalian models.

**Table 2:** Permeability (*P*_e_ 10^−6^ cm ⋅ s^−1^) in the PAMPA‐BBB assay of 14 commercial drugs and tested compounds and predictive penetration in the CNS.

Compound	Bibliography value^[33]^	Compound	Bibliography value^[33]^
Verapamil	16.0	25.9 ±0.8	
Testosterone	17.0	26.6±0.9	
Corticosterone	5.1	6.7±0.1	
Clonidine	5.3	6.5±0.06	
Ofloxacin	0.8	0.1±0.09	
Lomefloxacin	0.0	0.8±0.05	
Progesterone	9.3	14.7±0.3	
Promazine	8.8	13.8±0.4	
Imipramine	13.0	16.8±0.3	
Hydrocortisone	1.9	1.4±0.06	
Piroxicam	2.5	2.0±0.08	
Desipramine	12.0	17.8±0.2	
Cimetidine	0.0	0.7±0.02	
Norfloxacin	0.1	8.8±0.4	
**E**		2.2±0.1	CNS+/‐
**F**		10.9±0.4	CNS+
**H**		17.4±2.4	CNS+

### Evaluation of EHMT1 (GLP) activity by luminescence assay

Last, but not least, further characterization of the best candidates was performed assessing their selectivity. Thus, we tested them against EHMT1 (G9a‐like protein, GLP). Most remarkably, all of the new G9a inhibitors showed very low, almost non‐existent, specificity against EHMT1 compared to the well‐established **UNC0638** (Table 3). We found that **E** did not inhibit EHMT1 from to 0.01 μM 10 μM. In contrast, compounds **F** and **H** showed slight activity against EHMT1 at low doses. However, they presented a very low percentage, and furthermore this trend was not observed at high doses.

**Table 3:** Activity against of EHMT1 (GLP) measured by luminescence assay.

	% Inhibition of EHMT1			
Compound	0.01 μM	0.1 μM	1 μM	10 μM
**UNC0638**	2_±1	41_±4	66_±4	84_±1
**E**	2_±1	4_±2	3_±3	8_±3
**F**	26_±5	14_±3	4_±2	1_±2
**H**	14_±1	7_±1	4_±1	2_±1

## Conclusion

To sum up, we have identified new G9a inhibitors through *in silico* and *in vitro* screens, with different scaffolds to those previously published inhibitors, and more potent. **E**, **F**, and **H** were further studied in *in vivo* assays. Interestingly, our selected compounds were able to reduce H3 K9me2 levels similar to **UNC0638**, suggesting the G9a is the direct target for them. On the one hand, the effect on the impact of Aβ aggregates reduction was similar for **UNC0638** and **E**. Also, this compound showed promising pharmacokinetics results; however, its predicted BBB‐permeability was lower than **UNC0638**, possibly because of decreased lipophilicity due to lower logPo/w, logBB value, and higher PSA (Table S3). On the other hand, **H** did not show a positive effect related to Aβ deposition, albeit the *in silico* and *in vitro* results were promising, achieving the best predicted BBB permeability to date published for a G9a inhibitor. Nevertheless, it is noteworthy that compound **F** reduced Aβ deposition by approximately 40 %, thereby suggesting a promising role for this compound in AD pathology. Strikingly, **F** has an IC_50_=1.8 nM (Figure S2e), and none of the previously reported G9a inhibitors have such nanomolar efficacy. Besides, in comparison to the well‐established G9a inhibitor (**UNC0638**), **F** exhibited a higher permeability to BBB, as well as a higher selectivity for G9a, as it exhibited low activity against EHMT1. Therefore, these outcomes highlight an important difference and reinforce the potential use in neurodegenerative diseases of these novel G9a inhibitors. Hereinafter, future perspective will be to address the biological activity of our best candidate, **F** (5‐chloro‐N‐((3S,4S)‐1‐ethyl‐4‐methoxypyrrolidin‐3‐yl)‐1H‐indole‐2‐carboxamide) and its biological differentiation from other known G9a inhibitors. Then, our study will help future research establish the structure‐activity relationship (SAR) of **F** analogs to G9a, improving potency and BBB permeability as well as reducing one of the main AD hallmarks the Aβ plaques.

## Experimental Section

### Structure‐based virtual screening

PDB id of G9a for developing docking model was selected based on resolution, sequence length, PDB validation, and co‐crystallized molecule. The crystal structure of G9a bound to MS012 (Figure 1) (PDB ID: 5TTF) was retrieved from the RCSB portal.^[34]^ Refinement of protein was performed using a protein preparation wizard. The active site was defined around the co‐crystallized MS012 using the receptor grid generation module of Glide.^[35]^ The protocol resulting from a crystal structure (PDB ID: 5TTF) was validated using receiver operator characteristic (ROC) metrics and docking of co‐crystallized MS012 into the G9a (5TTF). The ROC analysis actives dataset was merged with the Schrodinger decoy set containing 1000 drug‐like decoy compounds.[^36^, ^37^] The combined dataset was docked into the protein using the SP docking method in glide. ROC plots were plotted using Enrichment Calculator in Maestro.^[38]^ A Chembridge CNS MPO library of 496.644 compounds was downloaded from the Chembridge portal were prepared with LigPrep using OPLS 2005 forcefield.^[35]^ The prepared Chembridge CNS MPO library was screened using virtual screening workflow docking protocols (HTVS, SP, and XP docking) of the Schrodinger software package on the selected crystal structure (PDB ID‐ 5TTF) carrying forward 10 % each from HTVS to SP and SP to XP and top 100 kept in XP docking. These selected top 100 molecules in XP docking were subjected to the binding affinity (dG) calculation using the Prime‐MM‐GBSA method. From these top 100 molecules, 9 molecules were selected based on the Glide XP docking score, dG of binding, nature of interaction with G9a active site key residues (Asp1074, Asp1083, and Asp1088),[^29^, ^34^, ^39^] and scaffold novelty.

### In silico analysis of CNS drugability

QikProp module of the Schrodinger software package was used for determining the drug properties. The 9 best scoring hits were taken for study resulting in basic physicochemical properties and *in silico* pharmacokinetic for CNS drugability.[^32^, ^40^]

### Compound preparation

G9a inhibitors were serially diluted between 5 nM and 0.001 nM in 100 % DMSO (Sigma, St. Louis, USA). Then, respective concentrations were subsequently diluted in MilliQ purified water to reach a final concentration ranging between 50 and 0.001 μM in 1 % DMSO in well.

### G9a homogenous assay

G9a activity was measured using the G9a Homogeneous Assay Kit (Catalog #52051, BPS Bioscience, San Diego, CA, USA). The G9a Homogeneous Assay Kit was set up in AlphaLISA® format on an optiplate‐384‐wells. Briefly, a sample containing the G9a enzyme is incubated with the biotinylated substrate plus inhibitor solution for two hours. Next, acceptor beads and primary antibody is added, followed by donor beads. Finally, the Alpha‐counts were read using an AlphaScreen microplate reader. The experiment was run per triplicate.

G9aactivity(%)=(C-Cb)/(Ct-Cb)x100

Ct=The chemiluminescence reading in the absence of the compound. The data set here is defined as 100 % activity in the absence of the compound.

Cb=The chemiluminescence reading in absence of enzyme. The data set here is defined as 0 % activity in the absence of the enzyme.

C=The chemiluminescence reading in the presence of the compound.

### C. elegans strains, and maintenance

The WT *C. elegans* strain (N2), and the transgenic CL2006 (dvIs2 [pCL12(unc‐54/human Aβ peptide 1–42 minigene)+rol‐6(su1006)]) provided by the *C. elegans* Genetic Center were used. Standard methods were used for culturing and observing *C. elegans*. N2 were propagated at 20 °C, while CL2006 worms were maintained at 16 °C in a temperature‐controlled incubator on solid nematode growth medium (NGM) seeded with Escherichia coli (E. coli) OP50 strain as a food source.

#### Food clearance assay

N2 (WT) worms were subjected to a drug assay in liquid format. Worms were grown with continuous shaking at 180 rpm at 20 °C for 7 days. Each well contained a final volume of 60 μL, comprising 25–30 animals in the larva 1 (L1) stage diluted in S‐medium solution, G9a inhibitor at the appropriate dose, and OP50 inactivated by freeze‐thaw cycles suspended in S‐medium complete solution to a final OD_595_ of 0.7 measured in the microplate reader. Tested concentrations ranged between 1 μM to 0.001 μM. For control wells were used DMSO 1 %(vehicle) and DMSO 5 % (toxic condition); and for blank wells were used S‐medium and S‐medium complete only, without eggs or OP50, respectively. The effect of compounds on *C. elegans* physiology was monitored by the rate at which the OP50 suspension was consumed, as a readout for *C. elegans* growth, survival, or fecundity. The OD_595_ was measured daily. Assays were run in triplicates (n=3), with a total of at least 150 animals tested per compound concentration.

#### Locomotion assay

Locomotion assay of G9a inhibitors was assessed to obtain dose‐response profile, evaluating the impact of the pharmacology treatment in motor dysfunction presented by the transgenic strains CL2006. Nematodes were synchronized by alkaline hypochlorite treatment, and drug assay was performed as described above, except for the OP50 bacteria OD_595_ adjusted to 0.9, and the duration of the treatment was 5 days. N2 (WT), and the transgenic strain CL2006 were treated in liquid media for 4 days at 20 °C and 180 rpm, starting at the L1 stage. On day 5 of age, worms were transferred from the 96‐well plates onto an unseeded NGM plate for 45 minutes before starting the trial, allowing the plates to dry and worms. Locomotion assays were performed in 30 mm NGM plates, in which the whole surface of the plate was covered by OP50. 5 to 10 adult nematodes were placed in the center of a circle (with 1 cm of diameter) in the seeded 30 mm NGM plates. After 1 min, the number of animals remaining inside the circle was scored as a locomotor defect. Motor behavior assays were run in triplicates (n=3), with a total of at least 100 animals tested per compound concentration. Motor index establishes a score where treated animals showing the same motor defect as CL2006 have an index of 0 %. By contrast, when animals improve motor behavior comparable to the WT (N2) they get an index of 100 %. All the results shown in the figures are calculated using the following formula:

$$\mathrm{Motor}\;\mathrm{index}\;\left(\%\right)=\left(\mathrm{LD}\;\mathrm{CL}2006{}_{\mathrm{vehicle}}\;-\;\mathrm{LD}\;\mathrm{CL}2006{}_{\mathrm{drug}}\right)\;/(\mathrm{LD}\;\mathrm{CL}2006{}_{\mathrm{vehicle}}\;-\;\mathrm{LD}\;\mathrm{WT}{}_{\mathrm{vehicle}})\;\times \;100$$

LD=locomotiondefective

#### Thioflavin‐S staining Aβ aggregation

After 5 days of treatment, adult CL2006 *C. elegans* were fixed in 4 % Paraformaldehyde/Phosphate‐buffered saline (PBS) (pH 7.5), for 24 hours at 4 °C. Then, worms were permeabilized in 5 % fresh β‐mercaptoethanol, 1 % Triton X‐100, 125 mm Tris (pH 7.5), at 37 °C for another 24 hours. On the last day, nematodes were stained with 0.125 % Thioflavin‐S (ThS) (Sigma, CAS# 1326–12‐1) in 50 % ethanol (EtOH) for 2 min, destained in 50 % EtOH for 2 min, washed 3 times with PBS. To prepare the glass slide for microscopy, approximately 10 μL volume was transferred on a droplet of Fluoromount G (Electron Microscopy Sciences, CAT#17984‐25). Fluorescence images were acquired using a 20 Å∼ objective of a fluorescence microscope. Aβ in the head region of worms were quantified by counting the number of Th−S positive spots using ImageJ and were expressed as Aβ deposits/anterior area. Aβ aggregates were scored by an investigator blinded to G9a inhibitors treatments.

### Western blotting

To determine the ratio of H3 K9me2/H3 total N2 (WT) and CL2006 animals were incubated in liquid culture with vehicle (DMSO 1 %), or 0.1 μM of G9a inhibitor in a 96‐well plate format as already mentioned. For chronic treatment, four‐day‐old animals were collected with M9 buffer. Histone extraction was performed following the manufacturer's instructions (EpiQuik Total Histone Extraction HT Kit, EpiGentek, #OP‐0007‐192). The samples were resolved in a 14 % SDS‐gel, as previously described.^[12]^ To capture chemiluminescence signals were used Amersham Imager 680 and Western blot quantifications were performed using ImageLab software (Bio‐Rad). Immunoblots were probed with anti‐H3 K9me2 (1 : 1000) (Epigentek, #A‐4035), and anti‐H3 total (1 : 1000) (Cell signaling, #9715).

#### PAMPA‐BBB assay

To evaluate the brain penetration of the different compounds, a parallel artificial membrane permeation assay for BBB was used, following the method described by Di et al..^[33]^ The *in vitro* P_e_ of fourteen commercial drugs through lipid extract of porcine brain membrane together with the test compounds were determined. Commercial drugs and assayed compounds were tested using a mixture of PBS:EtOH (70 : 30). Assay validation was made by comparing the experimental permeability with the reported values of the commercial drugs by bibliography, and linear correlation between experimental and reported permeability of the fourteen commercial drugs using the parallel artificial membrane permeation assay was evaluated (y=1.572x–1.147; R^2^=0.9452). From this equation and taking into account the limits established by Di et al.^[33]^ for BBB permeation, we established the ranges of permeability as compounds of high BBB permeation (CNS+): P_e_ (10^−6^ cm ⋅ s^−1^) >5.14; compounds of low BBB permeation(CNS‐): P_e_ (10^−6^ cm ⋅ s^−1^) <1.99 and compounds of uncertain BBB permeation(CNS+/−):5.14 Pe (10^−6^ cm ⋅ s^−1^) >1.99.

### EHMT1 (GLP) chemiluminescence assay kit

The assay was carried out using EHMT1 (GLP) Chemiluminescent Assay kit. Compounds were added on a 96‐well plate precoated with histone substrate supplied by kit. 30 μL of master mixture SAM were added and the reaction was initiated adding 20 μL of diluted EHMT1 (GLP) enzyme. The reaction was incubated at room temperature (RT) for 1 hour. The plate was washed three times with 200 μl TBST buffer. 100 μL of Blocking buffer were added to every well and the plate was incubated at RT for 10 minutes with shaking. Supernatant was removed and 100 μl of Primary antibody were added per well. The plate was incubated at RT for 1 hour with slow shaking. The plate was washed three times with 200 μl TBST buffer. 100 μL of Blocking buffer were added to every well and the plate was incubated at RT for 10 minutes with shaking. 100 μl of Secondary HRP‐labeled antibody were added per well and the plate was incubated at RT for 30 minutes with slow shaking. The plate was washed three times with 200 μl TBST buffer. Finally, 100 μl of substrate mix were added and the luminescence was measurement with integration time of 1 second in in Perkin Elmer EnSpire Multimode plate reader.

### Statistical analysis

Data analysis was conducted using GraphPad Prism ver. 9 statistical software. The statistical outliers were identified with Grubb's test and excluded from the analysis. Group comparisons were carried out with a One‐way Analysis of variance (ANOVA), followed by the Dunnett post hoc test. In some cases, comparison between groups was also performed by unpaired t‐test or One‐way ANOVA, followed by Fisher's LSD post hoc test. For the calculation of IC_50_, non‐linear regressions were performed. Regarding food clearance assay, results were compared non‐linear regression model for sigmoidal curves against the negative control. Values are shown as the mean ± Standard error of the mean (SEM) of at least n=3. Statistical significance was considered when p values were <0.05.

## Conflict of interest

The authors declare no conflict of interest.

1

## Supporting information

As a service to our authors and readers, this journal provides supporting information supplied by the authors. Such materials are peer reviewed and may be re‐organized for online delivery, but are not copy‐edited or typeset. Technical support issues arising from supporting information (other than missing files) should be addressed to the authors.

Supporting Information

## Data Availability

The data that support the findings of this study are available from the corresponding author upon reasonable request.
