# Supplementary material for: Structure‐Based Virtual Screening and in vitro and in vivo Analyses Revealed Potent Methyltransferase G9a Inhibitors as Prospective Anti‐Alzheimer's Agents
Source: ChemMedChem. 2022 May 19;17(13):e202200002. doi: 10.1002/cmdc.202200002 (PMC9401600; doi:10.1002/cmdc.202200002)
Supplement: Supplementary file 1 — Supporting Information [file CMDC-17-0-s001.pdf]

ChemMedChem

Supporting Information

Structure-Based Virtual Screening and *in vitro* and *in vivo* Analyses Revealed Potent Methyltransferase G9a Inhibitors as Prospective Anti-Alzheimer's Agents

Aina Bellver-Sanchis[+], Bhanwar Singh Choudhary[+], Júlia Companys-Alemany, Sukanya, Pedro A. Ávila-López, Antón Leandro Martínez Rodríguez, Jose Manuel Brea Floriani, Ruchi Malik, Mercè Pallàs, Belén Pérez, and Christian Griñán-Ferré*

Table of contents

Page S2	Figure S1: Superimposed co-crystallized and docked MS012.
Page S3	Table S1: 2D interaction diagram of selected screened molecules.
Page S4	Figure S2: IC_{50} curve for each compound tested Vs. well established G9a inhibitor, UNC0638.
Page S5	Table S2: Raw data of the percentage of G9a activity.
Page S6	Table S3: Predicted QikProp properties for CNS drugability.
Page S7	Figure S3: Toxicity of G9a inhibitors through the food clearance assay.
Page S8	Figure S4: Locomotor defect exhibited by N2 strain and the transgenic AD strain, CL2006. Dose-response profile of the excluded G9a inhibitors.
Page S9	Figure S5: 3D interaction diagram of a) A-G9a, b) B-G9a, c) C-G9a. d) D-G9a, e) G-G9a, and f) I-G9a.
Page S10-S18	LC-MS purity data for screened compounds.
Page S19	PAINS analysis results

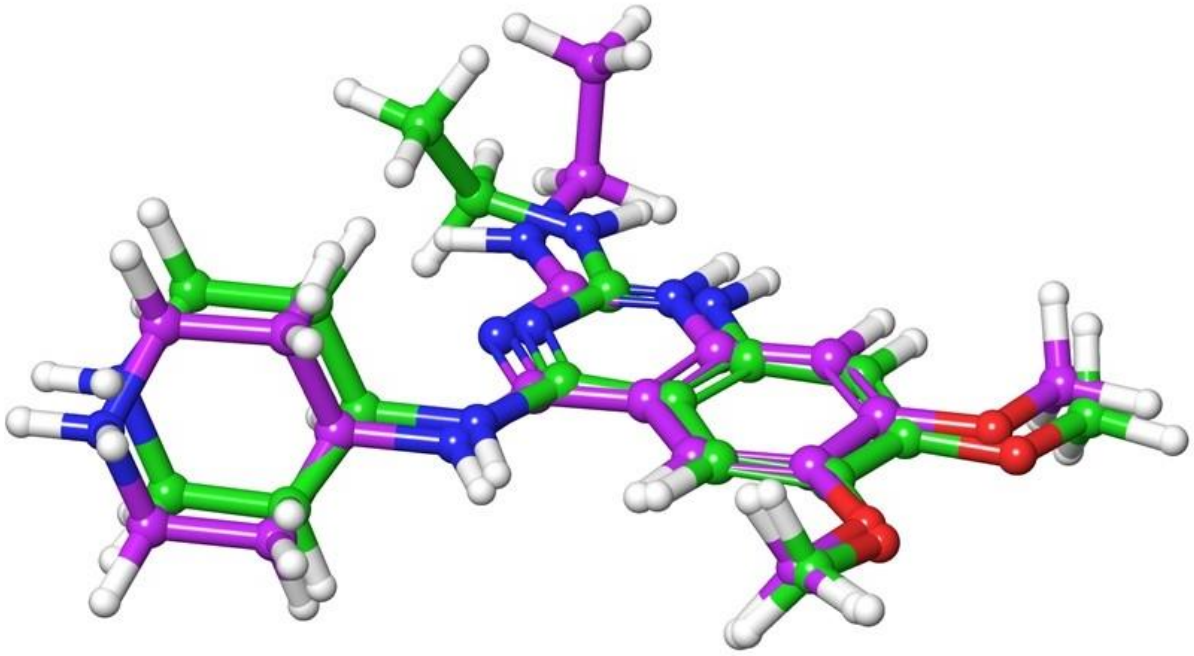

Figure S1. Superimposed co-crystallized (green) and docked (violet) MS012.

Table S1. 2D interaction diagram of selected screened molecules.

S. No.	ID	Code	Interaction type & Interacting residues
1	29352686	A	WB: Asp1083, Ser1084; π-π: Phe1087; π-cat: Tyr1154; SB: Asp1083; HBD: Asp1083, Leu1086
2	55607373	B	SB: Asp1074, Asp1078, Asp1083, Asp1088; π-π: Tyr1067, phe1087, Tyr1154; HBD: Asp1078, Ser1084, Leu1086
3	43631981	C	WB: Asp1083, Ser1084; π-π: Phe1087; SB: Asp1074, Asp1088; π-cat: Tyr1154, Arg1175, HBD: Asp1074, Asp1088, Leu1086
4	15589586	D	SB: Asp1082; HBD: Leu1086, Asp1088
5	55517816	E	SB: Asp1088; π-π: Phe1087; π-cat: Tyr1154; WB: Asp1083, Ser1084; HBD: Asp1074, Asp1078, Leu1086
6	31871543	F	WB: Asp1083, Ser1084; π-cat: Tyr1154; HBD: Leu1086, Asp1088
7	20839262	G	WB: Leu1086; HBD: Asp1078, Asp1088; SB: Asp1083
8	27379251	H	WB: Asp1083, Ser1084, Leu1086; SB: Asp1074, Asp1088; π-cat: Phe1087; π-π-: Phe1152; HBD: Leu1086, Asp1088, Tyr1154
9	12814353	I	WB: Asp1083, Ser1084; π-cat: Tyr1154; SB: Asp1083; HBD: Asp1083, Leu1086; HBA: Arg1157

WB: water bridge; SB: salt bridge; HBD: hydrogen bond donor; HBA: hydrogen bond acceptor; π-cat: π-cation interaction; π-π: π-π interaction; Asp: Aspartate; Leu: Leucine; Tyr: Tyrosine; Phe: Phenylalanine; Arg: Arginine; Ser: Serine.

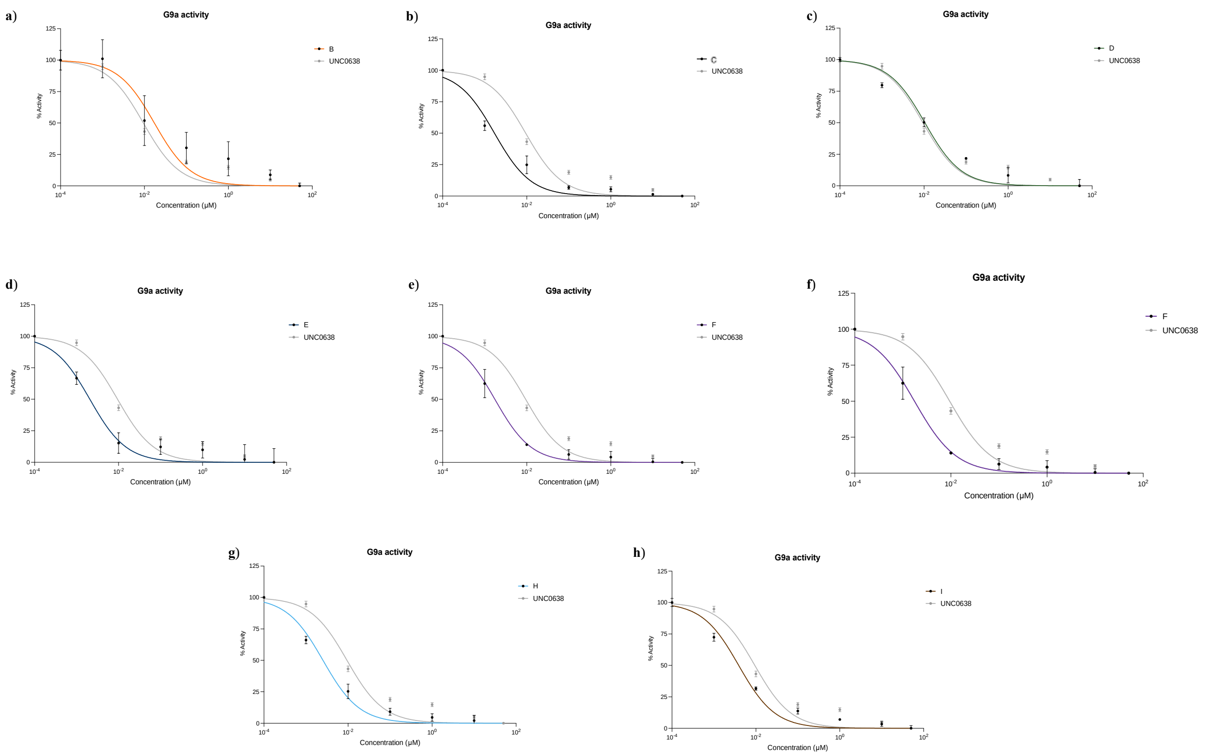

Figure S2. IC_{50} curve for each compound tested Vs. well established G9a inhibitor, UNC0638. (B: orange; C: black; D: green; E: navy blue; F: purple; G: pink; H: light blue; I: brown; UNC0638: grey.)

Table S2. Raw data of the percentage of G9a activity.

	% G9a activity									
µM	A	B	C	D	E	F	G	H	I	UNC0638
50	-0.4	0.2	10.7	9.9	10.3	12.3	7.7	4.2	5.9	0.1
10	10.0	0.9	10.9	8.3	10.6	12.8	8.5	4.9	6.6	1.9
1	14.8	1.2	11.5	11.5	13.7	13.8	8.7	6.1	6.8	5.2
0.1	22.8	2.1	12.0	15.8	14.4	14.0	11.4	7.6	8.2	7.9
0.01	22.9	3.5	15.1	23.0	14.6	14.7	17.6	11.8	13.5	17.0
0.001	27.8	8.7	22.2	29.3	30.3	24.6	26.0	27.8	24.0	34.1
0.0001	31.0	9.3	32.8	33.0	37.1	28.8	29.6	37.9	30.9	36.4

	% G9a activity									
µM	A	B	C	D	E	F	G	H	I	UNC0638
50	0.9	0.6	10.9	12.1	15.5	12.3	9.1	6.9	4.8	0.7
10	11.3	1.6	11.3	10.3	16.3	12.0	9.1	7.6	5.7	2.6
1	15.1	3.8	12.4	14.2	16.9	12.3	10.3	8.0	7.3	5.8
0.1	22.7	5.3	12.6	15.9	17.3	12.7	12.3	9.4	9.3	6.8
0.01	30.7	7.3	19.5	21.5	18.6	14.6	16.1	15.5	12.8	15.3
0.001	30.6	11.6	23.9	28.4	27.9	20.8	28.7	25.9	22.5	35.7
0.0001	32.1	10.8	32.7	33.8	37.3	29.0	33.7	37.5	29.3	37.2

	% G9a activity									
µM	A	B	C	D	E	F	G	H	I	UNC0638
50	0.3	0.4	10.8	11.0	12.9	12.3	8.4	5.6	5.3	0.3
10	10.6	1.2	11.1	9.3	13.5	12.4	8.8	6.2	6.2	1.9
1	15.0	2.5	12.0	12.8	15.3	13.0	9.5	7.0	7.1	6.3
0.1	22.7	3.7	12.3	15.9	15.9	13.4	11.9	8.5	8.7	7.0
0.01	26.8	5.4	17.3	22.2	16.6	14.6	16.8	13.7	13.2	16.1
0.001	29.2	10.1	23.1	28.8	29.1	22.7	27.4	26.8	23.2	34.9
0.0001	31.6	10.0	32.8	33.4	37.2	28.9	31.7	37.7	30.1	36.8

Table S3. Predicted QikProp properties for CNS drugability.

S. No.	Code	CNS	SASA	HBD	HBA	logPo/w	logS	PCaco	logBB	PMDCK	logKp	logKhsa	HOA	%HOA	PSA	Rule of 5
1	UNC0638	2	883.549	1	8	5.311	-5.371	413.395	-0.614	233.048	-4.919	1.165	2	78.956	46.568	2
2	A	1	654.894	1	9.2	2.142	-2.779	497.612	-0.064	257.398	-3.317	-0.222	3	87.754	72.923	0
3	B	1	696.62	0	7.25	3.363	-3.426	586.707	-0.085	307.553	-2.685	0.015	3	96.185	44.722	0
4	**C**	2	716.157	1	5	4.173	-3.768	342.913	0.643	344.802	-4.717	0.655	3	96.754	31.054	0
5	D	2	753.337	0	6.7	4.309	-3.408	476.039	0.844	436.6	-3.951	0.441	3	100	26.969	0
6	**E**	1	719.535	2	7	2.685	-3.675	85.439	0.169	69.055	-6.459	0.408	3	77.241	58.683	0
7	**F**	1	616.786	2	6.2	2.656	-3.717	617.29	0.249	799.747	-3.741	0.078	3	92.442	63.815	0
8	G	0	711.78	2	8.5	2.279	-3.618	233.856	-0.717	113.799	-3.928	-0.092	3	82.691	92.633	0
9	**H**	1	682.052	1	5.5	3.506	-4.117	611.772	0.306	895.879	-3.261	0.29	3	100	45.83	0
10	I	1	773.591	2	8.5	3.356	-4.817	356.15	-0.444	179.307	-3.807	0.359	3	92.265	88.391	0

*Cell denoted with red color fall outside the limits of CNS drugability.

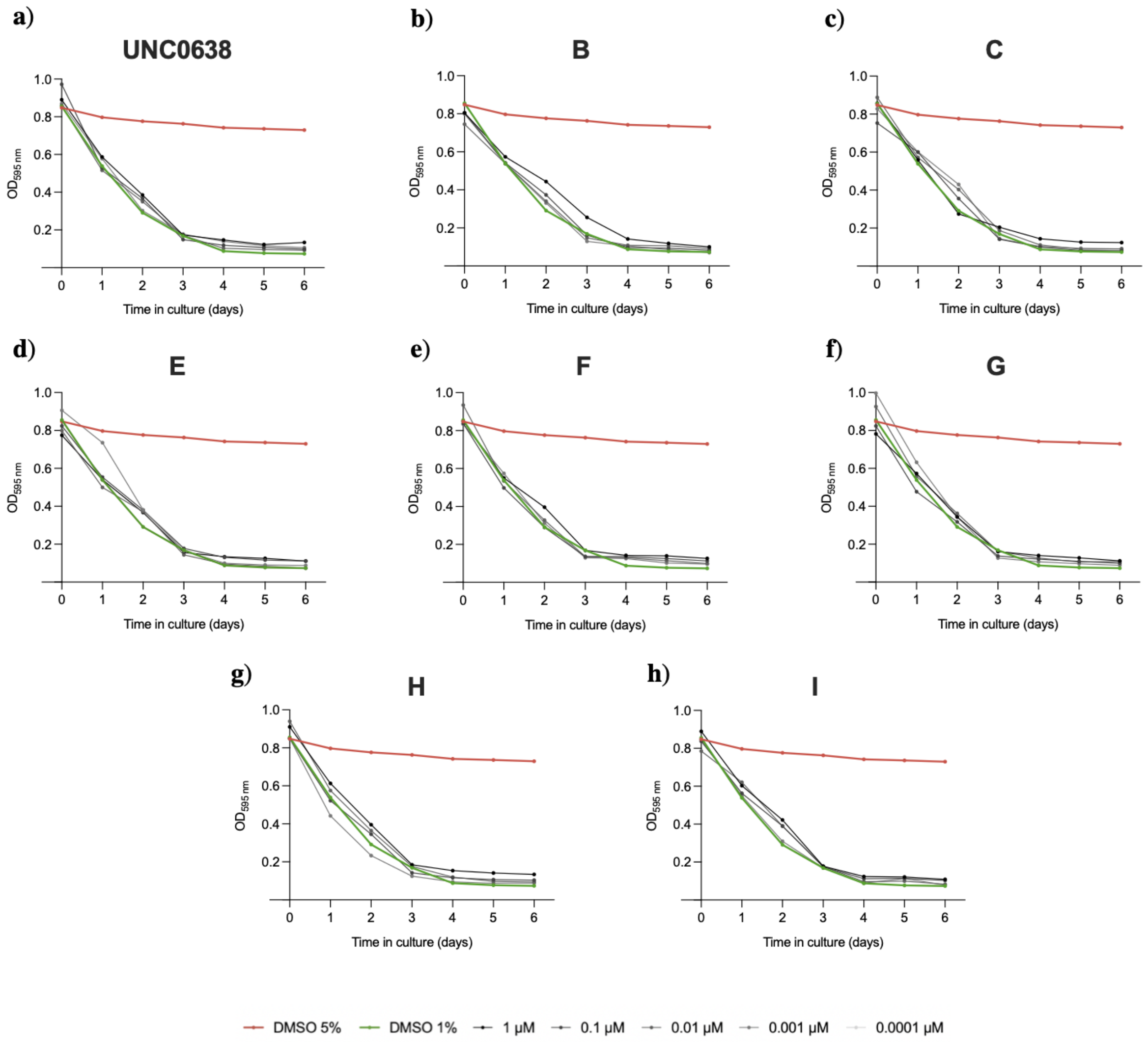

Figure S3. Toxicity of G9a inhibitors was performed through the food clearance assay. The optical density (OD_{595}) of *E. coli* OP50 suspension was measured daily in N2 *C. elegans* treated with the eight compounds in a dose range between 0.001 µM up to 1 µM (greyscale lines), starting at L1 stage. The mean OD_{595} was calculated for each day from six samples and plotted during the 6 days of the experiment. DMSO 5% (red line) was used as a positive control (toxic concentration); DMSO 1% (green line) was used as negative control (non-toxic concentratation), representing the normal development of the animals. Statistical analysis: Application of a non-linear regression model for sigmoidal curves against DMSO 1%.

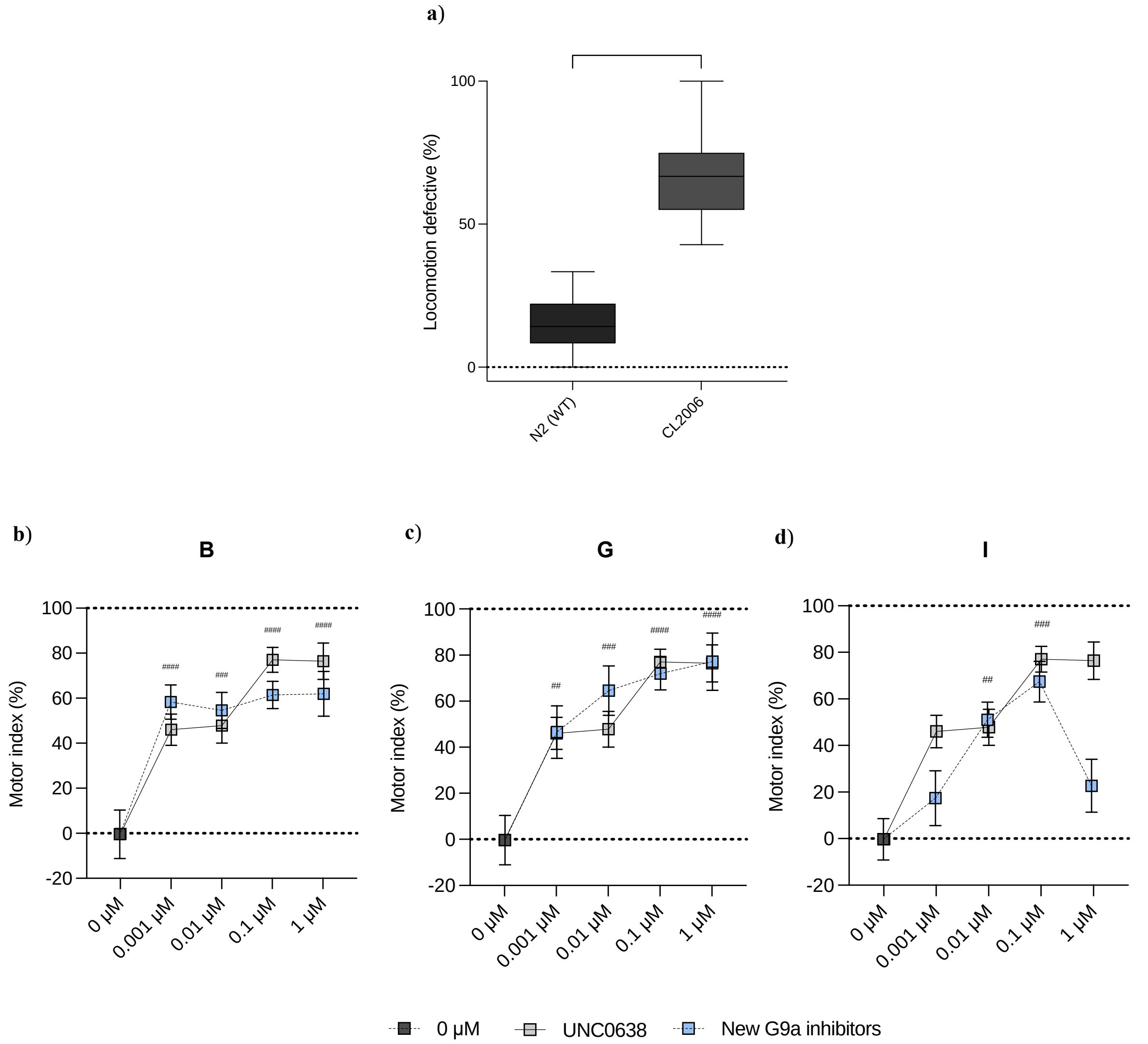

Figure S4. a) Representation for the locomotor defect exhibited by N2 strain and the transgenic AD strain, CL2006. b-d) Dose-response profile of the excluded G9a inhibitors**.** For the percent of locomotion defective: Values represented are mean ± Standard error of the mean (SEM); n = 3 with at least 90-100 worms in each group. Statistical analysis: One-Way ANOVA, followed by Dunnett post-hoc analysis. ****p<0.0001. For dose-response: Values represented are mean ± SEM; n = 3 with at least 90-100 worms in each group. Statistical analysis: One-Way ANOVA, followed by Dunnett post-hoc analysis. 0 µM Vs. UNC0638 treatment:***p<0.001; ****p<0.0001. 0 µM Vs. New G9a inhibitor treatment: $^{\#\#}p<0.01$; $^{\#\#\#}p<0.001$; $^{\#\#\#\#}p<0.0001$.

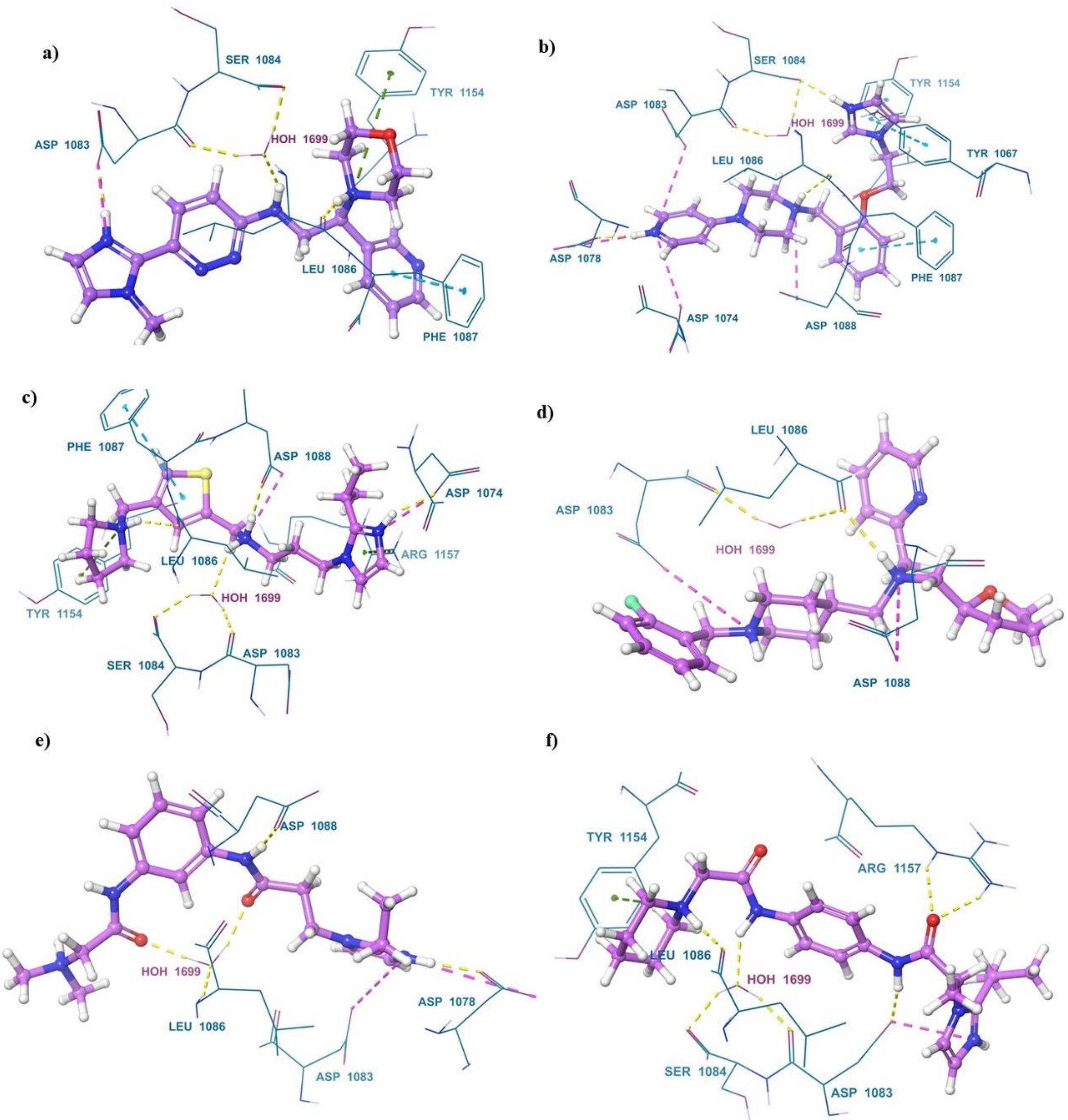

Figure S5. 3D interaction diagram of a) A-G9a, b) B-G9a, c) C-G9a, d) D-G9a, e) G-G9a and f) I-G9a. Protein-ligand Interactions were denoted by a dotted line. Hydrogen bond: Yellow; Salt bridge: Pink; π-π stacking: Blue; π-cation: Green.

FC94544722

A

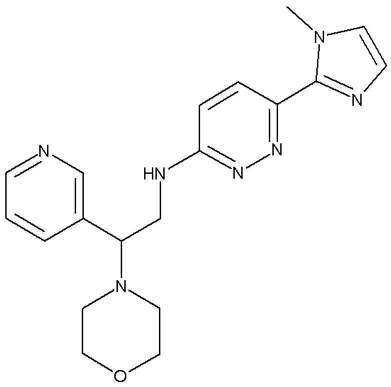

ID	29352686	365.4415	$C_{19}H_{23}N_7O$

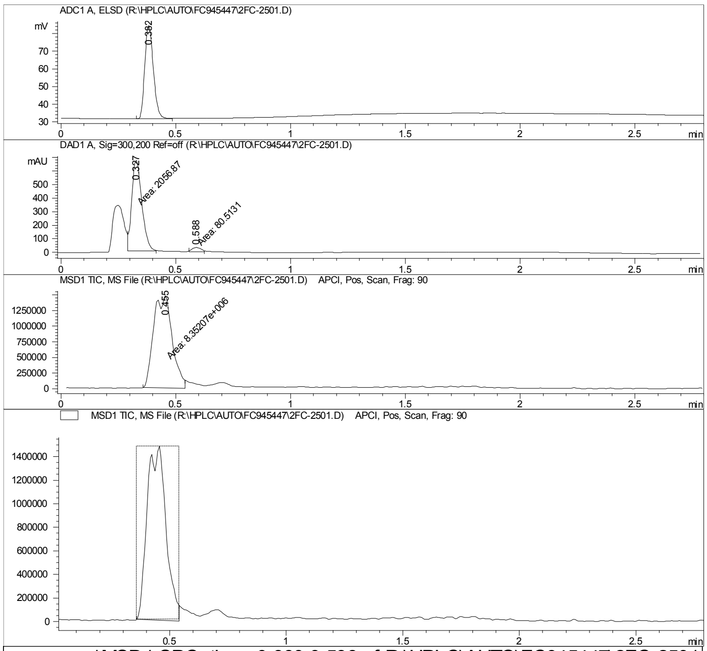

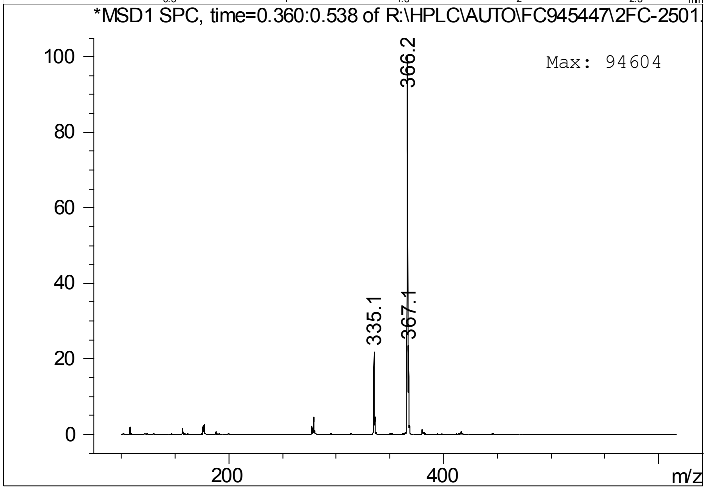


```
Data File R:\HPLC\AUTO\FC945447\2FC-2501.D
Sample Name: FC945447P2-F-03
Instrument 1 26/09/11 11:11:29
PMP1, Solvent A         : 0.1%TFA in Acn/H2O (2.5:97.5)
PMP1, Solvent B         : 0.1% TFA in AcN
PMP1, Solvent C         : 0.1%FA in ACN/H2O (2.5:97.5)
PMP1, Solvent D         : 0.1%FA in ACN
Ionization mode              :    APCI Positive

Signal 1: ADC1 A, ELSD
Peak RetTime Type  Width     Area      Height     Area
  #   [min]        [min]   [mV*s]      [mV]        %
----|-------|----|-------|----------|----------|--------|
   1   0.382 PB    0.0414  137.03101   52.63791 100.0000
Totals :                   137.03101   52.63791


Signal 2: DAD1 A, Sig=300,200 Ref=off
Peak RetTime Type  Width     Area      Height     Area
  #   [min]        [min]   [mAU*s]     [mAU]       %
----|-------|----|-------|----------|----------|--------|
   1   0.327 MM    0.0513 2056.87305  668.02240  96.2331
   2   0.588 MM    0.0455   80.51310   29.52247   3.7669
Totals :                  2137.38615  697.54487


Signal 3: MSD1 TIC, MS File
Peak RetTime Type  Width     Area      Height     Area
  #   [min]        [min]                           %
----|-------|----|-------|----------|----------|--------|
   1   0.455 MM    0.0937 8.35207e6  1.48519e6  100.0000
Totals :                  8.35207e6  1.48519e6
```

FC941590936 **B**

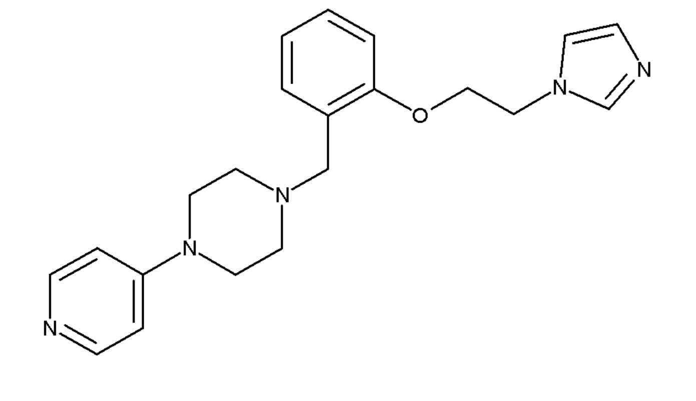

ID	55607373	363.4663	$C_{21}H_{25}N_5O$

Data File R:\HPLC\AUTO\1DE-3201.D
Sample Name: FC9415909P1-D-05
Instrument 1 05/08/2014 18:13:30
Column: Onyx C18 50x4.6mm | 3.75ml/min | Columns Reg Valve
Gradient: "A"->@2.2min->"B"(Hold 0.4min)->@0.2min->"A"->PostRun
PMP1, Solvent A : 0.1%TFA in Acn/H2O (2.5:97.5)
PMP1, Solvent B : 0.1%TFA in AcN
PMP1, Solvent C : 0.1%FA in Acn/H2O (2.5:97.5)
PMP1, Solvent D : 0.1%FA in AcN
Ionization mode : APCI Positive

Signal 1: ADC1 B, ELSD

Peak #	RetTime [min]	Type	Width [min]	Area [mAu*s]	Height [mAu]	Area %
1	0.754	MM	0.0184	1.80391	1.63314	1.2903
2	0.809	MF	0.0372	128.73773	57.72703	92.0813
3	0.879	FM	0.0614	9.26716	2.51536	6.6285
Totals :				139.80880	61.87553	

Signal 2: DAD1 A, Sig=300,200 Ref=off

Peak #	RetTime [min]	Type	Width [min]	Area [mAU*s]	Height [mAU]	Area %
1	0.699	MF	0.0394	26.17825	11.06329	1.5820
2	0.768	MF	0.0448	1550.68164	577.10406	93.7122
3	0.842	FM	0.0343	51.59457	25.05865	3.1180
4	1.041	MM	0.0306	26.27340	14.28967	1.5878
Totals :				1654.72787	627.51569	

Signal 3: MSD1 TIC, MS File

Peak #	RetTime [min]	Type	Width [min]	Area	Height	Area %
1	0.795	MM	0.0494	2.78428e6	9.40117e5	100.0000
Totals :				2.78428e6	9.40117e5	

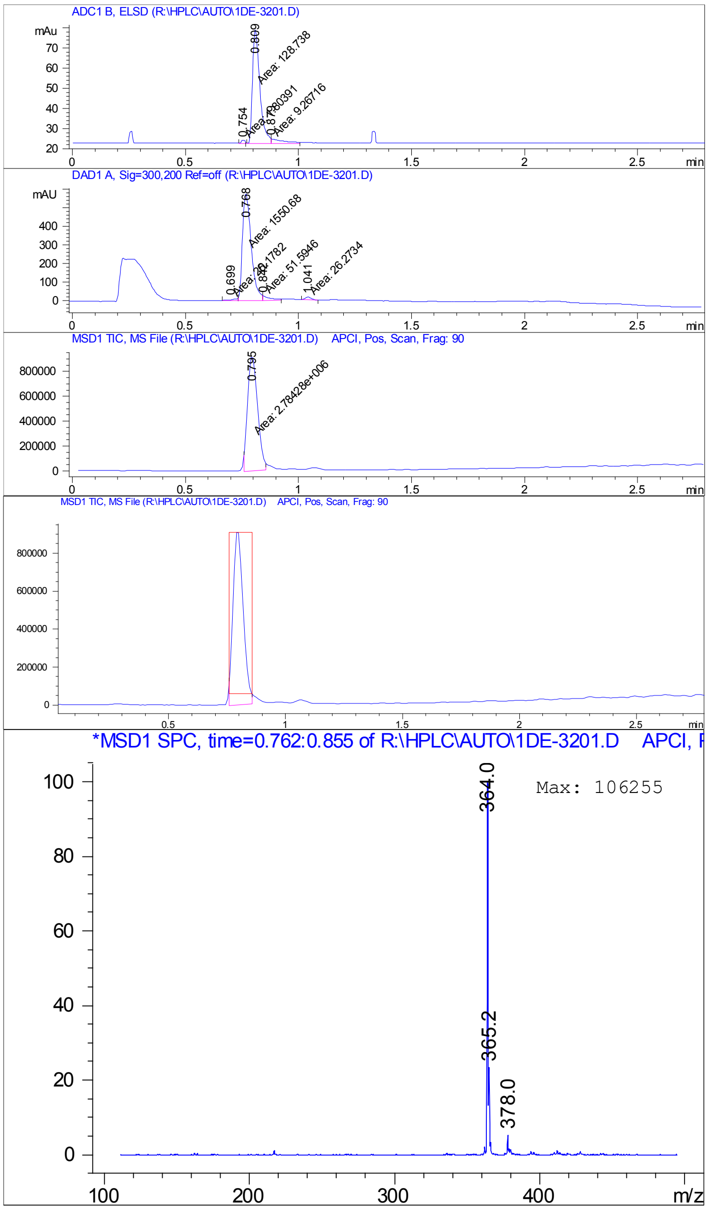

FC942332308 **C**

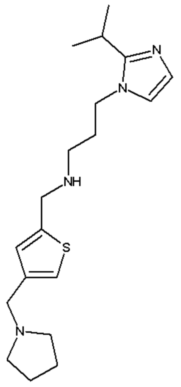

ID	43631981	346.5418	$C_{19}H_{30}N_4S$

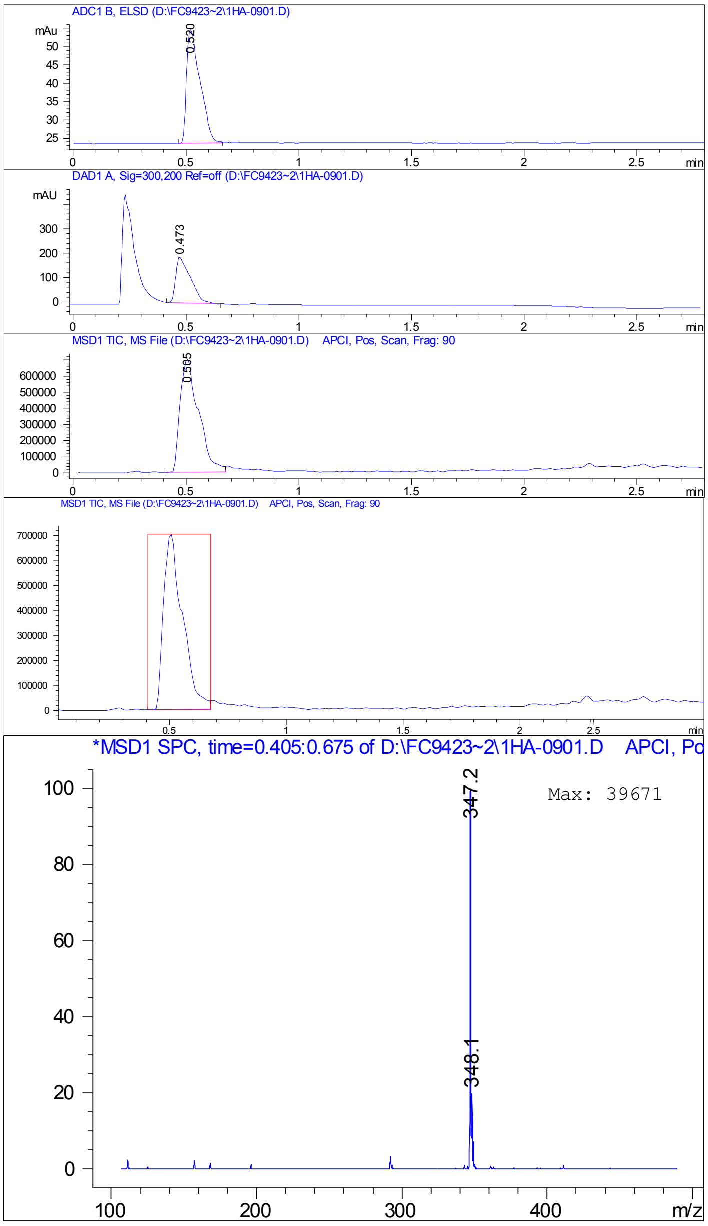


```
Data File D:\FC9423~2\1HA-0901.D
Sample Name: FC9423323P1-H-01
Instrument 1 18/09/2017 13:45:19
Column: Onyx C18 50x4.6mm | 3.75ml/min | Columns Reg Valve
Gradient: "A"->@2.0min->"B"(Hold 0.6min)->@0.2min->"A"->PostRun
PMP1, Solvent A          : 0.1%TFA in Acn/H2O (2.5:97.5)
PMP1, Solvent B          : 0.1%TFA in AcN
PMP1, Solvent C          : 0.1%FA in Acn/H2O (2.5:97.5)
PMP1, Solvent D          : 0.1%FA in AcN
Ionization mode              :    APCI Positive

Signal 1: ADC1 B, ELSD
Peak RetTime Type  Width      Area       Height      Area
  #    [min]        [min]    [mAu*s]     [mAu]         %
----|-------|----|-------|----------|----------|--------|
   1   0.520 PB    0.0605  128.23772   30.87906 100.0000
Totals :                   128.23772   30.87906


Signal 2: DAD1 A, Sig=300,200 Ref=off
Peak RetTime Type  Width      Area       Height      Area
  #    [min]        [min]    [mAU*s]     [mAU]         %
----|-------|----|-------|----------|----------|--------|
   1   0.473 PP    0.0650  836.74414  184.24127 100.0000
Totals :                   836.74414  184.24127


Signal 3: MSD1 TIC, MS File
Peak RetTime Type  Width      Area       Height      Area
  #    [min]        [min]                              %
----|-------|----|-------|----------|----------|--------|
   1   0.505 BB    0.0858 3.98199e6  7.01022e5  100.0000
Totals :                  3.98199e6  7.01022e5
```

PH10250647 **D**

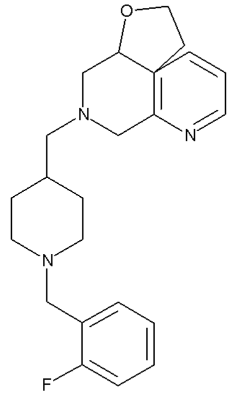

ID	15589586	397.5405	$C_{24}H_{32}FN_3O$

Data File C:\HPCHEM\1\DATA\424\2GF-9301.D
Sample Name: PH102506P2-G-06
Instrument 1 18/06/2006 07:45:38 lcms#6
Column: Hi-Q C18 5um 50x4.6mm | 3.75ml/min
Gradient: "A"->@2.4min->"B"(Hold 0.3min)->@0.1min->"A"->PostRun
PMP1, Solvent A : 0.1% TFA in AcN/Water (5:95)
PMP1, Solvent B : 0.1% TFA in AcN
PMP1, Solvent C : 10mM CH3COONH4 in AcN/Water (2.5:75.5)
PMP1, Solvent D : 10mM CH3COONH4 in AcN/Water (90:10)
Ionization mode : APCI Positive

Signal 1: ADC1 A, ELSD

Peak #	RetTime [min]	Type	Width [min]	Area [mV*s]	Height [mV]	Area %
1	1.092	BP	0.0534	20.30906	5.86045	100.0000
Totals :				20.30906	5.86045	

Signal 2: DAD1 A, Sig=260,2 Ref=off

Peak #	RetTime [min]	Type	Width [min]	Area [mAU*s]	Height [mAU]	Area %
1	0.779	BV	0.0631	214.16794	50.87011	3.3699
2	0.875	VV	0.0558	171.88681	45.80094	2.7046
3	1.053	VV	0.0606	5036.19385	1261.80518	79.2433
4	1.188	VV	0.0529	201.90080	54.88534	3.1769
5	1.288	VV	0.0782	257.65500	46.79729	4.0541
6	1.379	VB	0.1439	473.55078	41.89930	7.4512
Totals :				6355.35518	1502.05817	

Signal 3: MSD1 TIC, MS File

Peak #	RetTime [min]	Type	Width [min]	Area	Height	Area %
1	1.125	PB	0.0963	6.47657e7	1.01304e7	100.0000
Totals :				6.47657e7	1.01304e7	

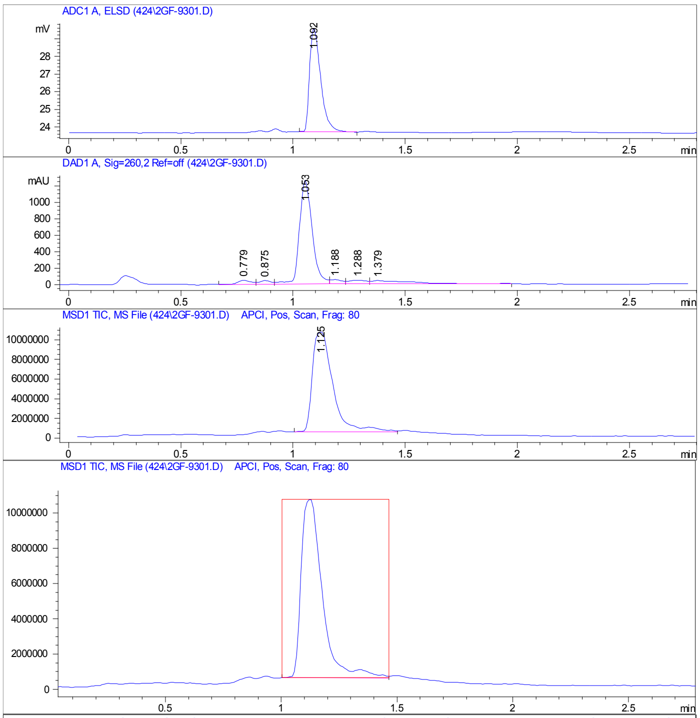

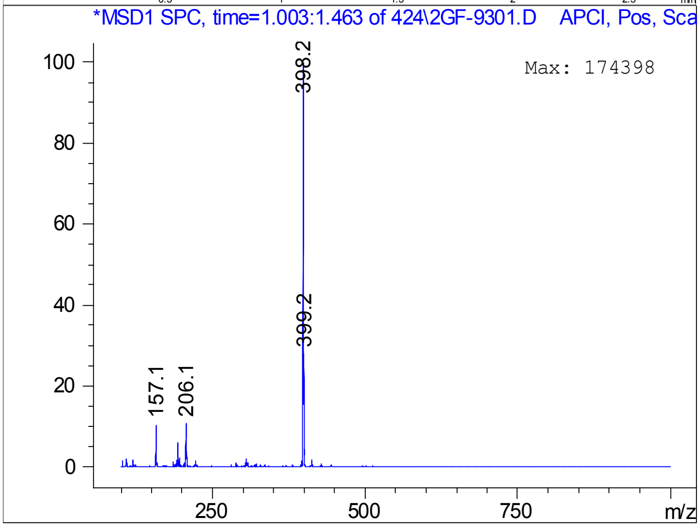

FC942237731

E

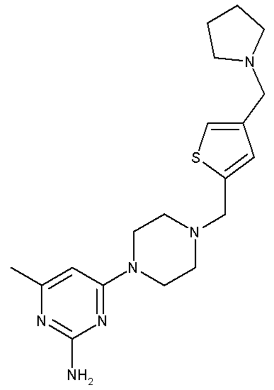

ID	55517816	372.5392	$C_{19}H_{28}N_6S$

Data File D:\FC9422~2\2GD-7101.D
Sample Name: FC9422377P2-G-04
Instrument 1 19/05/2017 12:01:10
Column: Onyx C18 50x4.6mm | 3.75ml/min | Columns Reg Valve
Gradient: "A"->@2.2min->"B"(Hold 0.4min)->@0.2min->"A"->PostRun
PMP1, Solvent A : 0.1%TFA, 2.5%AcN in H2O
PMP1, Solvent B : 0.1%TFA in AcN
PMP1, Solvent C : --NOT USED--
PMP1, Solvent D : --NOT USED--
Ionization mode : API-ES Positive

Signal 1: ADC1 B, ELSD

Peak #	RetTime [min]	Type	Width [min]	Area [mV*s]	Height [mV]	Area %
1	0.830	BB	0.0338	155.88422	69.21435	100.0000
Totals :				155.88422	69.21435	

Signal 2: DAD1 A, Sig=300,200 Ref=off

Peak #	RetTime [min]	Type	Width [min]	Area [mAU*s]	Height [mAU]	Area %
1	0.728	BB	0.0258	1182.20801	697.45557	100.0000
Totals :				1182.20801	697.45557	

Signal 3: MSD1 TIC, MS File

Peak #	RetTime [min]	Type	Width [min]	Area	Height	Area %
1	0.771	PB	0.0552	1.45094e6	4.20406e5	100.0000
Totals :				1.45094e6	4.20406e5	

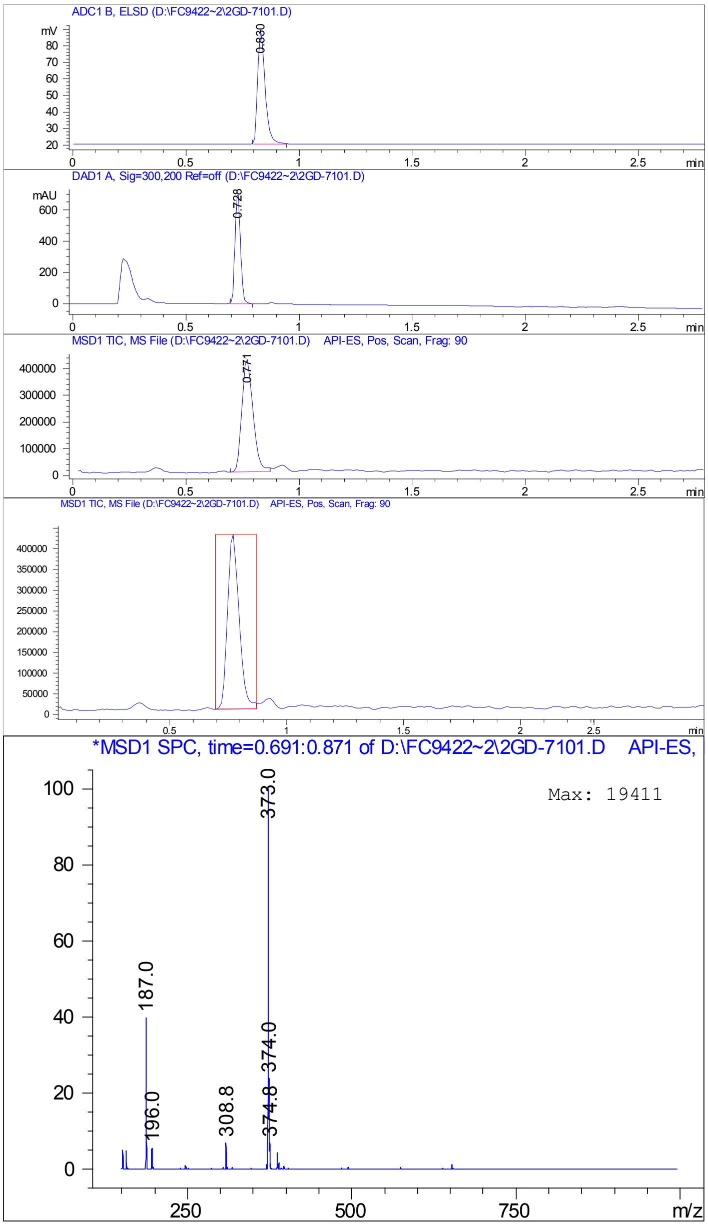

FC941705836 **F**

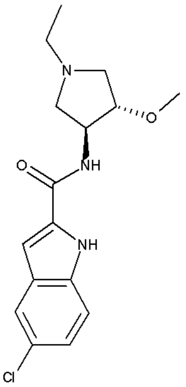

ID	31871543	321.8097	$C_{16}H_{20}ClN_3O_2$

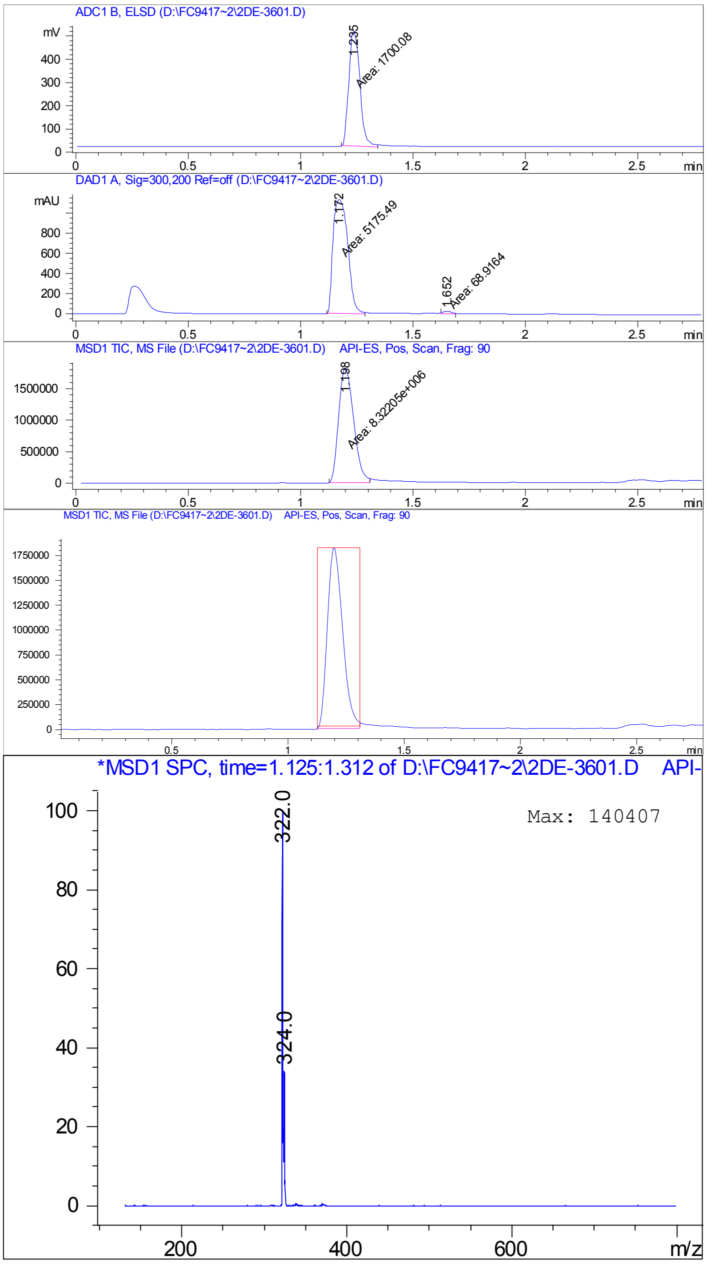

Data File D:\FC9417~2\2DE-3601.D
Sample Name: FC9417058P2-D-05
Instrument 1 07/05/2015 10:41:39
Column: Onyx C18 50x4.6mm | 3.75ml/min | Columns Reg Valve
Gradient: "A"->@2.2min->"B"(Hold 0.4min)->@0.2min->"A"->PostRun
PMP1, Solvent A : 0.1%TFA, 2.5%AcN/W
PMP1, Solvent B : 0.1%TFA/AcN
PMP1, Solvent C : --NOT USED--
PMP1, Solvent D : --NOT USED--
Ionization mode : API-ES Positive

Signal 1: ADC1 B, ELSD

Peak #	RetTime [min]	Type	Width [min]	Area [mV*s]	Height [mV]	Area %
1	1.235	MM	0.0569	1700.08313	498.27438	100.0000
Totals :				1700.08313	498.27438	

Signal 2: DAD1 A, Sig=300,200 Ref=off

Peak #	RetTime [min]	Type	Width [min]	Area [mAU*s]	Height [mAU]	Area %
1	1.172	MM	0.0760	5175.48926	1135.61267	98.6859
2	1.652	MM	0.0454	68.91638	25.30288	1.3141
Totals :				5244.40564	1160.91555	

Signal 3: MSD1 TIC, MS File

Peak #	RetTime [min]	Type	Width [min]	Area	Height	Area %
1	1.198	MM	0.0754	8.32205e6	1.84024e6	100.0000
Totals :				8.32205e6	1.84024e6	

FC942323248

G

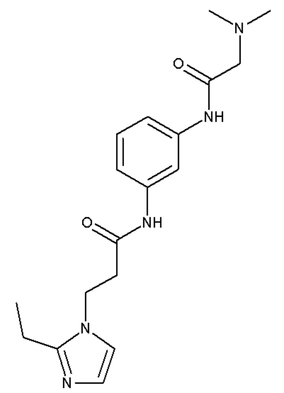

ID	20839262	343.4323	$C_{18}H_{25}N_5O_2$

```
Data File D:\FC9423~1\2HF-4701.D
Sample Name: FC9423232P2-H-06
Instrument 1 04/09/2017 13:58:00
Column: Onyx C18 50x4.6mm | 3.75ml/min | Columns Reg Valve
Gradient: "A"->@2.0min->"B"(Hold 0.6min)->@0.2min->"A"->PostRun
PMP1, Solvent A          : 0.1%TFA in Acn/H2O (2.5:97.5)
PMP1, Solvent B          : 0.1%TFA in AcN
PMP1, Solvent C          : 0.1%FA in Acn/H2O (2.5:97.5)
PMP1, Solvent D          : 0.1%FA in AcN
Ionization mode              :    APCI Positive

Signal 1: ADC1 B, ELSD
Peak RetTime Type  Width     Area       Height     Area
  #   [min]        [min]   [mAu*s]     [mAu]        %
----|-------|----|-------|----------|----------|--------|
   1   0.648 MM    0.0345   42.82569   20.67672  96.5118
   2   0.784 MM    0.0473    1.54785 5.45230e-1   3.4882
Totals :                    44.37354   21.22195


Signal 2: DAD1 A, Sig=300,200 Ref=off
Peak RetTime Type  Width     Area       Height     Area
  #   [min]        [min]   [mAU*s]     [mAU]        %
----|-------|----|-------|----------|----------|--------|
   1   0.598 PB    0.0346  840.42780  397.98636  84.6199
   2   0.696 BB    0.0433  152.75237   47.80603  15.3801
Totals :                   993.18016  445.79239


Signal 3: MSD1 TIC, MS File
Peak RetTime Type  Width     Area       Height     Area
  #   [min]        [min]                            %
----|-------|----|-------|----------|----------|--------|
   1   0.626 MM    0.0456 1.04022e6  3.80027e5  100.0000
Totals :                  1.04022e6  3.80027e5
```

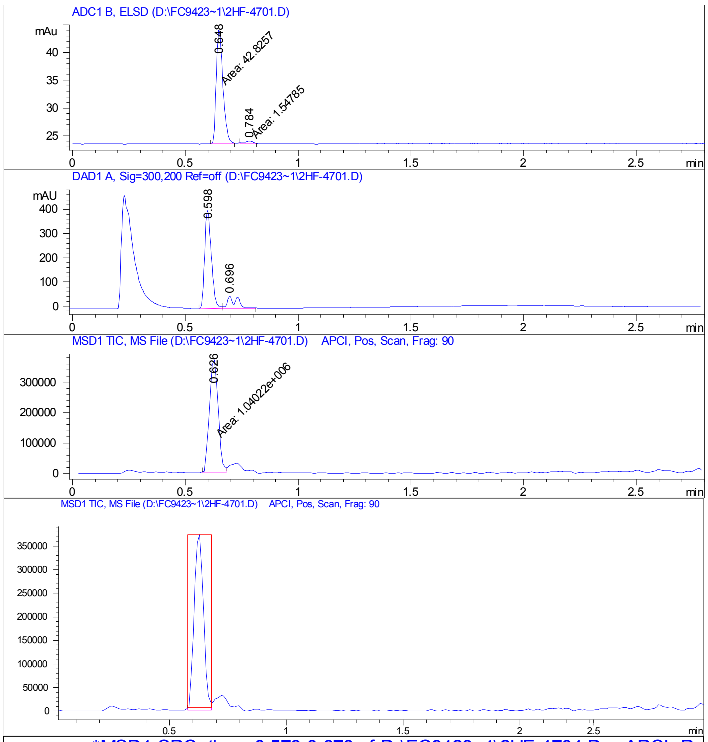

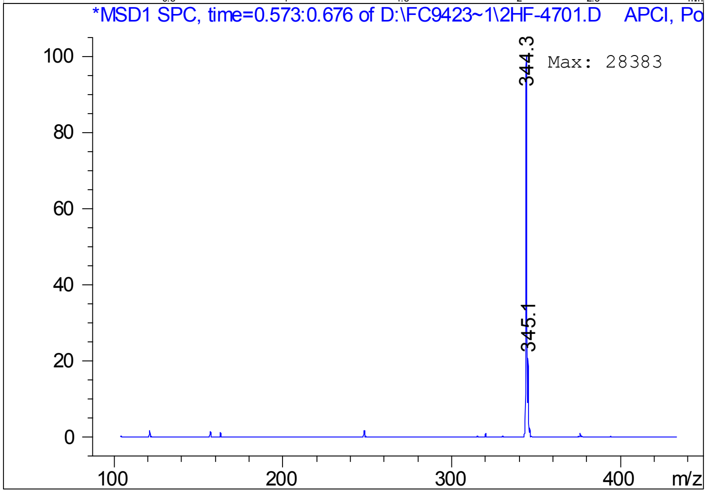

FC942342903 **H**

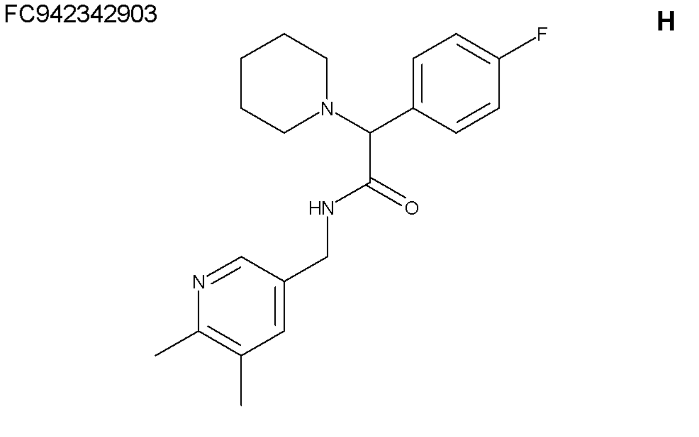

ID 27379251	355.4593	$C_{21}H_{26}FN_3O$

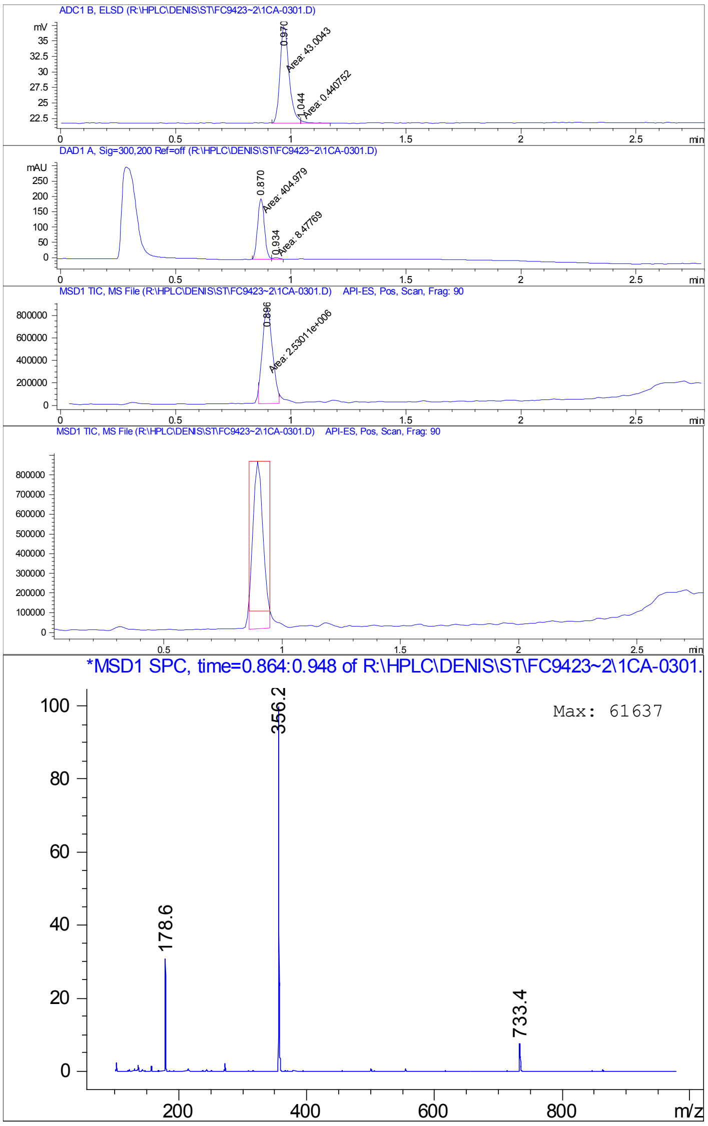


```
Data File R:\HPLC\DENIS\ST\FC9423~2\1CA-0301.D
Sample Name: FC9423429P1-C-01
Instrument 1 29/09/2017 13:25:04
Column: Onyx C18 50x4.6mm | 3.75ml/min | Columns Reg Valve
Gradient: "A"->@2.2min->"B"(Hold 0.4min)->@0.2min->"A"->PostRun
PMP1, Solvent A          : 0.1%TFA, 2.5%AcN in H2O
PMP1, Solvent B          : 0.1%TFA in AcN
PMP1, Solvent C          : --NOT USED--
PMP1, Solvent D          : --NOT USED--
Ionization mode              :    API-ES Positive

Signal 1: ADC1 B, ELSD
Peak RetTime Type  Width     Area       Height     Area
  #   [min]        [min]    [mV*s]      [mV]         %
----|-------|----|-------|----------|----------|--------|
   1   0.970 MF    0.0449   43.00430   15.96386   98.9855
   2   1.044 FM    0.0214 4.40752e-1 3.43719e-1    1.0145
Totals :                    43.44505   16.30758

Signal 2: DAD1 A, Sig=300,200 Ref=off
Peak RetTime Type  Width     Area       Height     Area
  #   [min]        [min]   [mAU*s]     [mAU]         %
----|-------|----|-------|----------|----------|--------|
   1   0.870 MF    0.0342  404.97916  197.24895   97.9496
   2   0.934 FM    0.0312    8.47769    4.52932    2.0504
Totals :                   413.45684  201.77827

Signal 3: MSD1 TIC, MS File
Peak RetTime Type  Width     Area       Height     Area
  #   [min]        [min]                             %
----|-------|----|-------|----------|----------|--------|
   1   0.896 MM    0.0491 2.53011e6  8.59311e5  100.0000
Totals :                  2.53011e6  8.59311e5
```

FC942284317

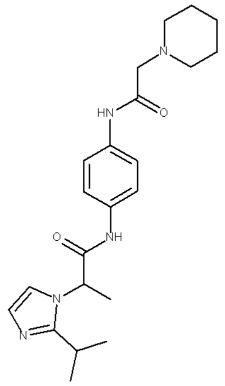

I

ID	12814353	397.5247	$C_{22}H_{31}N_5O_2$

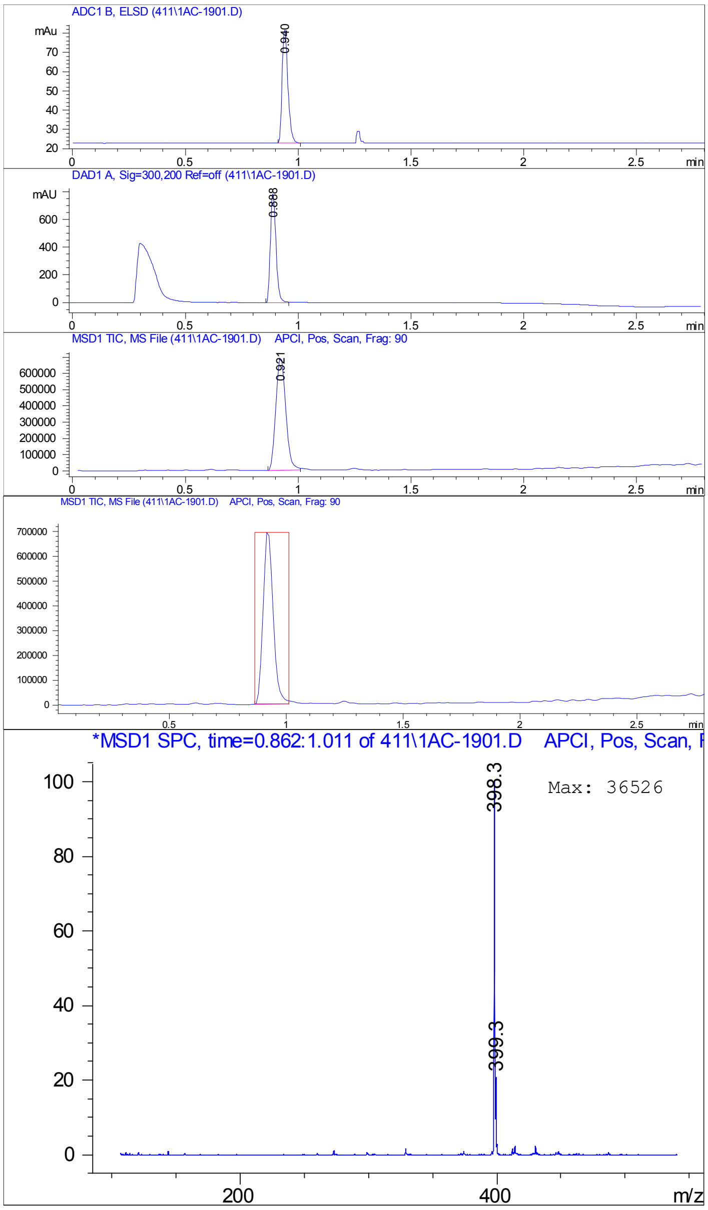


```
Data File D:\DATA\411\1AC-1901.D
Sample Name: FC9422843P1-A-03
Instrument 1 14/07/2017 15:47:48  N6
Column: Onyx Monolithic C18 50x4.6mm | 3.75ml/min | Columns Reg
Valve
Gradient: "A"->@2.2min->"B"(Hold 0.4min)->@0.2min->"A"->PostRun
PMP1, Solvent A          : 0.1%TFA in Acn/H2O (2.5:97.5)
PMP1, Solvent B          : 0.1%TFA in AcN
PMP1, Solvent C          : 0.1%FA in Acn/H2O (2.5:97.5)
PMP1, Solvent D          : 0.1%FA in AcN
Ionization mode              :    APCI Positive

Signal 1: ADC1 B, ELSD
Peak RetTime Type  Width     Area      Height     Area
  #   [min]        [min]   [mAu*s]     [mAu]        %
----|-------|----|-------|----------|----------|--------|
   1   0.940 BB    0.0277  104.46929   58.95369 100.0000
Totals :                   104.46929   58.95369


Signal 2: DAD1 A, Sig=300,200 Ref=off
Peak RetTime Type  Width     Area      Height     Area
  #   [min]        [min]   [mAU*s]     [mAU]        %
----|-------|----|-------|----------|----------|--------|
   1   0.888 PB    0.0284 1351.10828  772.64844 100.0000
Totals :                  1351.10828  772.64844


Signal 3: MSD1 TIC, MS File
Peak RetTime Type  Width     Area      Height     Area
  #   [min]        [min]                            %
----|-------|----|-------|----------|----------|--------|
   1   0.921 PB    0.0461 2.10541e6  7.03687e5  100.0000
Totals :                  2.10541e6  7.03687e5
```

False Positive Remover

Welcome Created 12-2009

Main Page>>Retrieve Structure

Return

Totally 9 Compound(s) passed the filter.Download (Smiles format)
The following are the structure(s) of compound(s) that passed the filter

736748.sdf 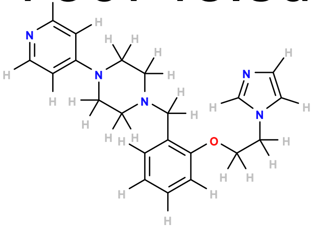	736748.sdf 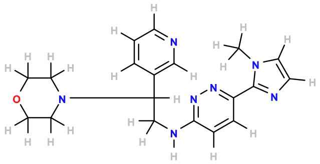	736748.sdf 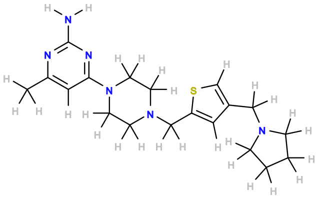	736748.sdf 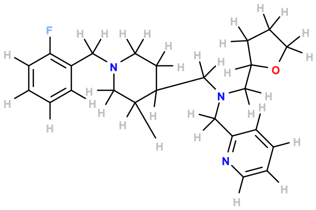
736748.sdf 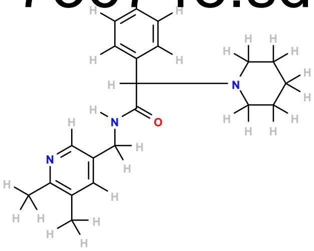	736748.sdf 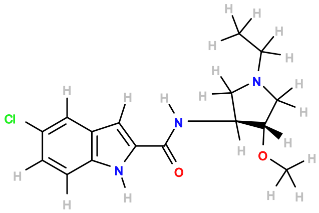	736748.sdf 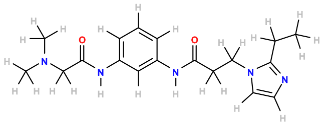	736748.sdf 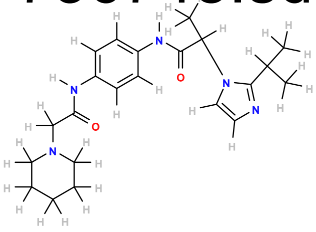
736748.sdf 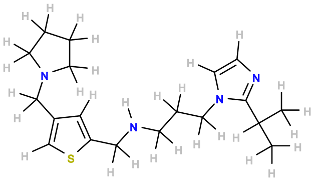			

Return

1
